# Loss of mGlu_5_ receptors in somatostatin-expressing neurons alters negative emotional states

**DOI:** 10.1038/s41380-024-02541-5

**Published:** 2024-04-04

**Authors:** Arnau Ramos-Prats, Pawel Matulewicz, Marie-Luise Edenhofer, Kai-Yi Wang, Chia-Wei Yeh, Ana Fajardo-Serrano, Michaela Kress, Kai Kummer, Cheng-Chang Lien, Francesco Ferraguti

**Affiliations:** 1 Institute of Pharmacology, Medical University of Innsbruck, Innsbruck, Austria; 2 Institute of Physiology, Medical University of Innsbruck, Innsbruck, Austria; 3 Institute of Neuroscience, National Yang Ming Chiao Tung University, Taipei, Taiwan; 4 Present Address: Friedrich Miescher Institute for Biomedical Research, Basel, Switzerland

**Keywords:** Neuroscience, Psychiatric disorders

## Abstract

Subtype 5 metabotropic glutamate receptors (mGlu_5_) are known to play an important role in regulating cognitive, social and valence systems. However, it remains largely unknown at which circuits and neuronal types mGlu_5_ act to influence these behavioral domains. Altered tissue- or cell-specific expression or function of mGlu_5_ has been proposed to contribute to the exacerbation of neuropsychiatric disorders. Here, we examined how these receptors regulate the activity of somatostatin-expressing (SST+) neurons, as well as their influence on behavior and brain rhythmic activity. Loss of mGlu_5_ in SST+ neurons elicited excitatory synaptic dysfunction in a region and sex-specific manner together with a range of emotional imbalances including diminished social novelty preference, reduced anxiety-like behavior and decreased freezing during retrieval of fear memories. In addition, the absence of mGlu_5_ in SST+ neurons during fear processing impaired theta frequency oscillatory activity in the medial prefrontal cortex and ventral hippocampus. These findings reveal a critical role of mGlu_5_ in controlling SST+ neurons excitability necessary for regulating negative emotional states.

## Introduction

Affective and anxiety disorders represent together one of the leading causes of disability worldwide [1]. Despite their enormous impact on public health, they have remained largely refractory to the identification of their causal risk factors and fundamental neurobiology. These disorders show a high degree of comorbidity [2] and lack clear genetic and biological boundaries [3, 4], thus possibly sharing common mechanistic features. They are characterized by shared emotional state abnormalities that may result from a dysfunction in the balance between cortical GABAergic and glutamatergic systems [5, 6]. Deficits in the expression or function of metabotropic glutamate type 5 receptors (mGlu_5_) have been proposed as possible mechanisms underlying this imbalance [7–9]. These receptors contribute to a number of important activity-dependent synaptic changes, such as long-term potentiation (LTP) and depression (LTD) at many central synapses [10, 11]. They also regulate *N*-Methyl-*D*-Aspartate (NMDA) glutamate receptor activity and neuronal excitability [12–14]. The distribution of mGlu_5_ is mostly observed in telencephalic areas, with highest expression levels in the hippocampus (HPC), striatum, lateral septum (LS) and prefrontal cortex (PFC) [15–17] not only in glutamatergic neurons, but also in GABAergic interneurons (INs) and glial cells [18–21].

So far, relatively few studies have investigated how mGlu_5_ in specific neuronal populations and brain circuits influence behavior and could thus contribute to altered cognitive and emotional processing across psychiatric disorders. Depletion of mGlu_5_ in glutamatergic principal neurons throughout all cortical regions in mice did not affect anxiety-like behavior, fear-learning, sensorimotor gating or social interaction, but resulted in increased novelty-induced locomotion and dysfunctional stress coping [22]. Conversely, deletion of mGlu_5_ in forebrain GABAergic neurons gave rise to a complex phenotype mostly characterized by changes in the regulation of locomotion and habituation responses [23], as well as to reduced anxiety-like behavior as shown by lower novelty-suppressed feeding and better coping to unescapable stress [24]. These consequences were only marginally recapitulated by the genetic ablation of mGlu_5_ in parvalbumin-expressing (PV+) cells. Interestingly, these animals displayed a broad repertoire of memory deficits, including social and non-social memory, but displayed intact anxiety-like behavior [25].

In recent years, somatostatin-expressing (SST+) neurons have gained increased attention with respect to affective and anxiety disorders [26], since several studies reported a crucial role played by these neurons in fear, anxiety and aversive learning in the PFC [27], anterior cingulate cortex [28], LS [29], HPC [30], amygdala [31] and other brain structures. SST+ neurons are the second largest group of INs in the cortex after PV+ INs, where they constitute about 1–3% of the overall neuronal population. They are widely present in the whole rodent brain except for the cerebellum and are highly heterogeneous [32]. For instance, SST expression can be found in multipolar, bipolar and fusiform cells, in Martinotti, basket, double-bouquet and bi-tufted neurons [32–34] and their electrophysiological properties range from low-threshold to burst-accommodating, burst-irregular spiking and non-accommodating firing responses [32, 35]. In the cortex (neocortex, basolateral amygdala and HPC), SST+ INs express mGlu_5_ [19–21], though the specific neuron subclasses remain largely unidentified. Little is also known regarding the expression of this receptor in SST+ cells of subcortical brain regions, such as the central amygdala (CeA) and LS.

Most SST+ INs target the distal dendrites of pyramidal neurons (PNs), where they gate dendritic excitability to provide feedback inhibition [36]. Moreover, SST+ INs strongly facilitate brain oscillatory activity [37], mostly in the theta range (3-12 Hz) [38] that is believed to integrate information from different brain regions [39] and has been linked to the processing of emotional cues [38, 40]. Thus, dysfunctional activity of SST+ INs at different cortical circuits may cause behavioral abnormalities of distinctive nature such as domain-specific emotional or cognitive deficits [26].

We hypothesized that mGlu_5_ expression in SST+ neurons regulates their activity and in turn controls emotional behaviors. To test this hypothesis, we selectively deleted mGlu_5_ in SST+ neurons in mice and explored behavioral phenotypes related to social, cognitive and emotional domains. Moreover, we evaluated the effects on local neuronal activity and assessed brain oscillatory activity in the medial PFC (mPFC) and ventral hippocampus (vHPC) during aversive emotional processing.

## Materials and methods

### Animals

All procedures involving animals were approved by the Austrian Animal Experimentation Ethics Board (license 2020-0.547.574 and 66.011/0141-V/3b/2019) and by the Institutional Animal Care and Use Committee of the National Yang Ming Chiao Tung University. They were performed in compliance with the European Convention for the Protection of Vertebrate Animals used for Experimental and Other Scientific Purposes (ETS no. 123). Every effort was taken to minimize the number of animals used.

C57BL/6 J mice were obtained from Charles River (Sulzfeld, Germany) or the Taiwan National Laboratory Animal Center. *Grm5*^Flox/Flox^ mice were kindly provided by Dr. Contractor (Northwestern University, Chicago) [41]. Sst-IRES-Cre (#013044 and #018973) and Ai14 (#007914) mice were obtained from Jackson Laboratories.

Sst-IRES-Cre (SST^Cre^) mice were cross-bred with *Grm5*^Flox/Flox^ mice, on a C57BL/6 J background to obtain deletion of mGlu_5_ specifically in SST neurons. Animals heterozygous for the Sst-IRES-Cre allele and homozygous for the floxed *Grm5* were used for all experiments (Suppl. Fig. 3a). In order to avoid possible deficits in maternal care, breeding was carried out using SST^Cre^ negative females and Cre positive males. Animals were weaned at 4 weeks of age and group-housed in a climate-controlled facility on a 12 h/12 h light/dark cycle with lights on at 07:00 AM, with water and food *ad libitum*. Genotyping was performed from ear punches and determined by PCR.

### Immunofluorescence experiments

For immunofluorescence staining, mice were deeply anaesthetized with thiopental sodium (150 mg/kg, i.p.) and transcardially perfused with a fixative as previously described [42]. Following brain extraction, coronal sections were cut (50 μm) on a Leica VT1000S vibratome (Leica Microsystems, Vienna, Austria) and immunostained against mGlu_5_ and other cellular markers based on previously described procedures [43]. Extended methods are provided as supplementary material.

### Pre-embedding immuno-electron microscopy

For immuno-electron microscopy, 0.05% glutaraldehyde was included in the fixative. Immunocytochemistry for electron microscopy was performed as previously described [42] and using similar conditions as used for the immunofluorescence experiments, but omitting Triton X-100 from the buffers. Extended methods are provided as supplementary material.

### Slice preparation and patch-clamp recording

**Fig. 1:**
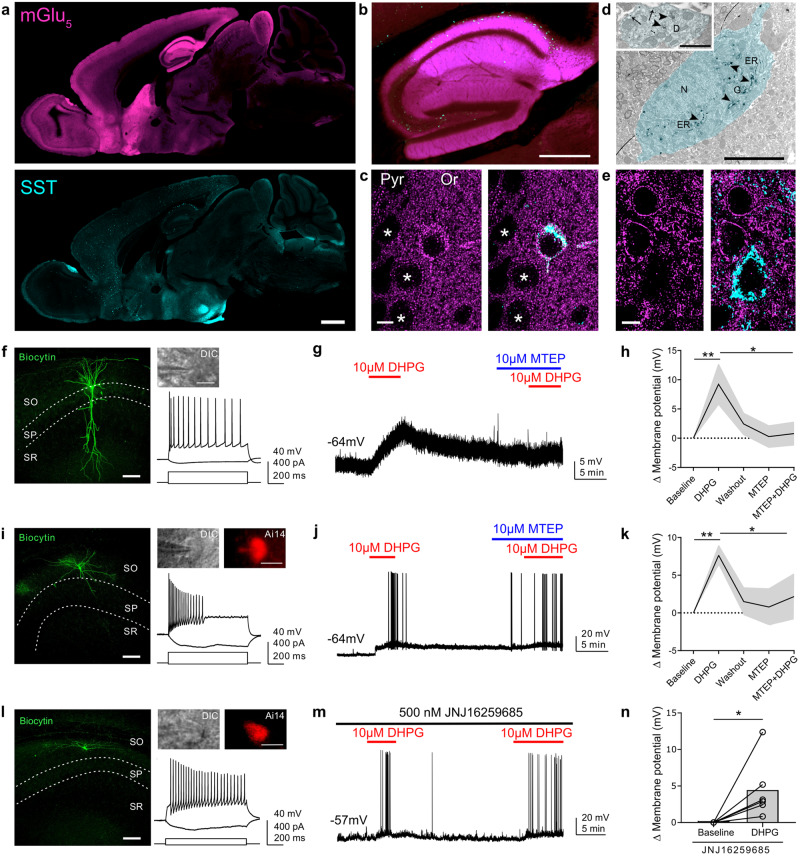
Anatomical and functional evidence of the co-expression between mGlu_5_ and SST. **a** Sagittal section of a WT mouse brain immunoreactive for mGlu_5_ (top) and SST (bottom). Scale bar: 1 mm. **b** Higher magnification of the hippocampus. Scale bar: 500 μm. **c** Representative image of mGlu_5_ (in magenta) and SST (turquoise) co-expression in an IN in the stratum oriens (Or) of the mouse HPC. Of note it is the high intracytoplasmic immunofluorescence signal for mGlu_5_ in comparison to pyramidal neurons (marked with *), that show mostly labeling associated with the plasma membrane. Scale bar: 5 μm. **d** Electron micrograph of a CA1 oriens/alveus IN characterized by strong SST immunoreactivity (DAB electron-dense deposits indicated by arrowheads) in the Golgi and ER. The inset shows a dendrite of this IN, in which most of the immunometal particles identifying mGlu_5_ are intracytoplasmatic rather than associated with the plasma membrane (arrows). Abbreviations: D, dendrite; ER, endoplasmic reticulum; G, Golgi apparatus; N, nucleus. Scale bar: 5 μm; scale bar inset: 1 μm. **e** Example image of mGlu_5_ and SST expression in the mouse LS depicting the absence of co-localization. Scale bar: 5 μm. The thickness of the confocal z-stacks: **c** 5.02 μm; **e** 3.6 μm. **f** Representative example of a biocytin-filled PN (scale bar: 100 μm) with its respective DIC image (scale bar: 10 μm) and membrane responses to current injections. SO, stratum oriens; SP, stratum pyramidale; SR, stratum radiatum. **g** Example trace displaying PN membrane depolarization upon DHPG application and inhibition by MTEP pre-treatment. **h** Quantification of DHPG-mediated membrane potential change in PNs in the absence or presence of MTEP (*n* = 5). One-way RM ANOVA, Treatment *F*_4,16_ = 4.29, *p < 0.05. Bonferroni multiple comparisons test: *p < 0.05, **p < 0.01. Plot represents mean ± SEM. **i** Example image of a biocytin-filled CA1 stratum oriens SST+ IN with its respective DIC, Ai14-tdTomato expression and membrane responses to current injections. **j** Example trace displaying the membrane depolarization upon DHPG application and inhibition by MTEP pre-treatment in the same SST+ IN shown in **i**. **k** Quantification of DHPG-mediated membrane potential change in CA1 SST+ INs in the absence or presence of MTEP (*n* = 6). One-way RM ANOVA, Treatment *F*_4,20_ = 4.75, **p < 0.01. Bonferroni multiple comparisons test: *p < 0.05, **p < 0.01. Plot represents mean ± SEM. **l** Example image of a biocytin-filled CA1 SST+ IN with its respective DIC, Ai14-tdTomato expression, and membrane responses to current injections. **m** Example trace displaying the membrane depolarization upon DHPG infusion of the same SST+ IN shown in **l** in the presence of the mGlu_1_ NAM JNJ16259685. **n** Quantification of DHPG-mediated membrane potential change in hippocampal SST+ INs in the presence of JNJ16259685 (*n* = 6). Wilcoxon matched-pairs rank test, *W* = 21, *p < 0.05. Data are shown as mean ± SEM; individual data points are also provided. *p < 0.05.

SST^Cre^::Ai14 transgenic (2–3 month-old) and SST^Cre^-*Grm*5^−/−^ (3–4 month-old; Suppl. Fig. 4) mice were sacrificed by rapid decapitation under isoflurane anesthesia. Brains were mounted on the slicing chamber with oxygenated (95% O_2_ and 5% CO_2_) ice-cold sucrose saline containing (in mM): 87 NaCl, 25 NaHCO_3_, 1.25 NaH_2_PO_4_, 2.5 KCl, 10 glucose, 75 sucrose, 0.5 CaCl_2_, and 7 MgCl_2_. Acute horizontal brain slices, 350 µm thick, were cut using a vibratome (DTK-1000; Dosaka, Kyoto, Japan) and allowed to recover in oxygenated sucrose saline at 34 °C for 30 min and then kept at room temperature until use. For the ex vivo whole-cell patch-clamp recordings (Fig. 1f–n and Suppl. figs 2 and 4), slices were transferred to a submerged chamber and perfused at room temperature (23 ± 2 °C) with oxygenated artificial cerebrospinal fluid (aCSF) containing (in mM): 125 NaCl, 25 NaHCO_3_, 1.25 NaH_2_PO_4_, 2.5 KCl, 25 glucose, 2 CaCl_2_, and 1 MgCl_2_. The tdTomato and GFP expression in neurons was confirmed by epifluorescence. To examine the effect of mGlu_5_ activation on SST+ neurons, the group I mGlu agonist (S)-3,5-dihydroxyphenylglycine (DHPG; 10 μM, Tocris Bioscience), in the presence or not of the negative allosteric modulator (NAM) 3-((2-Methyl-1,3-thiazol-4-yl)ethynyl)pyridine (MTEP; 10 μM, Tocris Bioscience), was applied together with synaptic blockers [CNQX (10 μM), Tocris Bioscience; gabazine (1 μM), Abcam; CGP55845 (1 μM), Tocris Bioscience] via bath superfusion. In some experiments, 3,4-dihydro-2H-pyrano[(2,3-b])quinolin-7-yl)-(cis-4-methoxycyclohexyl)-methanone (JNJ16259685) (500 nM; Tocris Bioscience) was bath-applied to block mGlu_1_ and followed by DHPG application.

Whole-cell patch-clamp recordings were performed using the Axopatch 200B amplifier or Multiclamp 700B amplifier (Molecular Devices, San Jose, CA, USA). Recording electrodes (3–7 MΩ) were filled with a low Cl^−^ internal solution, which contained (in mM): 136.8 K-gluconate, 7.2 KCl, 0.2 EGTA, 4 MgATP, 10 HEPES, 7 Na_2_-phosphocreatine, 0.5 Na_3_GTP (pH 7.3 with KOH), and 0.4% biocytin (wt/vol, Thermo Fisher Scientific, Waltham, MA, USA). The pipette capacitance was compensated in the cell-attached mode. The series resistance was compensated to 100% in the current-clamp configuration. Signals were low-pass filtered at 4 kHz (four-pole Bessel filter) and sampled at 10 kHz using a digitizer (Digidata 1440 A). Pulse sequences were generated by pClamp 10.7 (Molecular Devices).

#### Multielectrode Array (MEA) excitability recordings

Brain slices were prepared from 12-17 week old mice as previously described [44]. The animals were anesthetized with isoflurane (IsoFlo®, Zoetis) and decapitated. Brains were rapidly removed and immersed in ice-cold aCSF. Coronal slices (300 µm) containing the hippocampal areas were cut in sucrose aCSF. The slices were incubated in oxygenated sucrose aCSF at 32 °C for 30 min and subsequently transferred to oxygenated standard aCSF.

Multi-electrode array (MEA) recordings were performed using a MEA2100 recording system as previously described [45]. The slices were transferred to planar MEA chips (120MEA200/30iR-Ti, Multi-Channel Systems, Reutlingen, Germany) and a platinum slice grid spanned with nylon fibers was carefully placed on top of the slices. After a short habituation period, spontaneous action potential firing was recorded with a sampling rate of 5 kHz for 5 min. Some slices were pre-treated with the mGlu_5_ NAM MTEP (10 µM) and/or mGlu_1_ NAM JNJ16259685 (1 µM) for 10 min. For stimulation the non-selective group I mGlu agonist DHPG (50 µM) was bath-applied for 2 min. All data streams were filtered using a 200 Hz high-pass filter. The recorded traces were analyzed using the MC_Rack software (MultiChannel Systems). Spike threshold was set to −5 standard deviations (SD) from baseline. Recording electrodes were classified as active if the firing frequency exceeded 1/60 Hz (i.e., at least one AP per min). Average firing frequencies were calculated for the different pharmacological treatments.

#### MEA LTP recordings

For LTP recordings, only male mice were used. Electrical stimuli were applied and the strength of the pulse was adapted to obtain field excitatory postsynaptic potentials (fEPSPs) with 50% maximal slope. An initial input output (IO) curve with increasing amplitudes from 500 mV to 4 V was generated using the MC_Stimulus II software. Schaffer collaterals were stimulated in the CA3 hippocampal region using 2–3 electrodes and evoked fEPSPs were recorded in the CA1 area. Biphasic constant pulses (0.1 ms/phase) were delivered every 60 s until a stable baseline was recorded for 15 min. LTP was induced by a theta burst stimulation (TBS) protocol (3 trains of 10 bursts at 5 Hz, 4 pulses at 100 Hz for each burst). Afterwards, test pulses were constantly delivered every 60 s for 40 min. The recordings were analyzed using the MC_Rack software. In order to quantify LTP, the fEPSP amplitudes after TBS stimulation were normalized to fEPSP amplitudes during baseline recording.

### Animal behavioral assays

All behavioral tests were performed by experimenters blinded to the genotype of the animals.

#### Social preference and social novelty

Social behavior was assessed by means of a modified three-chambered social task apparatus as previously described [43, 46]. Briefly, the procedure involved two phases: social preference and social novelty, each lasting 10 min. Mice were tested in the dark (infrared light; Lux < 5). After a 10 min habituation period, mice were temporarily contained in the center chamber by closing the side walls, and a novel young unfamiliar mouse (5–8 weeks) was placed into a mesh cylinder (15 cm tall, 7 cm diameter) in the least explored side chamber, whereas an identical empty mesh cylinder was placed in the opposite chamber. The test mouse was then allowed to explore the chambers for 10 min (social preference). On the second phase of the test, mice were again contained in the center chamber while another novel young unfamiliar mouse was placed in the chamber that previously contained the empty mesh cylinder, and test mice were allowed to explore the chambers for 10 min (social novelty). Time spent in interaction with the mesh cylinders (<5 cm) was automatically tracked and scored using the Ethovision XT12 software (Noldus; RRID:SCR_000441).

#### Elevated plus maze

Mice were allowed to explore an elevated platform (72 cm above the floor) consisting of two opposing open (30 × 5 cm) and two opposing closed arms (30 × 5 cm) for a total of 5 min. Illumination in the open arms was set at 50 Lux. Mice were placed individually in one of the closed arms. The behavior of each mouse was tracked with Ethovision XT12 software. Arm entries were defined as crossing of the center of mass of the animal. Measurements during the test included: time spent in the open arms, entries and distance traveled. The position of the animal within the maze was automatically tracked and scored using Ethovision XT12 software.

#### Fear conditioning and extinction

Mice were fear-conditioned in a 25 x 25 x 40 cm chamber (Ugo Basile, Comerio, Italy) with transparent walls and a metal grid floor for delivering a foot-shock. After 120 s acclimation period, an 80 dB 6 kHz noise [conditioned stimulus (CS)] lasting 30 s was paired in the last 2 s with a 0.6 mA scrambled foot-shock [unconditioned stimulus (US)] 5 times (120 s inter-pairing interval). Mice were returned to the home cage after 120 s no-stimulus consolidation period. The chamber was cleaned between subjects with 70% ethanol. Fear extinction training sessions were performed 24 h after the conditioning in a novel context with different visual (squared and striped patterned walls) and olfactory cues (1% acetic acid) and consisted of a 120 s baseline period, after which the CS was presented 20 times with a randomized inter-CS interval ranging from 5–40 s. The first three tone presentations on day 2 were taken as an indicator of fear retrieval. Freezing was automatically measured by ANY-maze software (Stoelting Europe, Dublin, Ireland) using a freezing minimum duration threshold of 1 s, and manually cross-checked by an experienced experimenter.

### Surgeries and electrophysiological recordings in vivo

Adult male mice were stereotactically implanted under ketamine/xylazine (i.p.) and sevoflurane (Sevorane, AbbVie GmbH, Austria) anesthesia with recording electrodes made of twisted 76,2 µm teflon coated, stainless steel wires (Science Products, Hofheim, Germany) into the mPFC (at 3° angle, AP: +1.8, L: +0.5, D:−1.7 mm—from bregma) and vHPC (AP:−3.2, L: + 3.3, D: −2.8 mm from bregma). A silver wire was used as a ground/reference electrode. All electrodes were connected to a 10-pin PCB connector and cemented to the skull with dental acrylic. Following recovery from surgery, mice were fear conditioned in a 25 × 25 × 40 cm chamber (Ugo Basile) with 5 US-CS pairing blocks consisting of a 15 s CS presentation (white noise) followed by a 1 s US foot-shock (0.5 mA) with 1 min inter-trial interval. Fear retrieval was performed 24 h in the same chamber where mice were subjected to fear conditioning but omitting the US. LFP signals were recorded during the retrieval phase on an EXT-9 recording system using a head-stage-commutator assembly (NPI electronic, Germany), allowing the animal to free movement inside the fear conditioning chamber. The raw signal was amplified, filtered, digitized, and stored on a PC for offline analysis with the use of Spike-2 (ver. 8.08) software (CED, Cambridge, UK).

Local field potentials (LFP) recorded from mPFC and vHPC during fear retrieval sessions were digitally filtered and used for further analysis. For spectral analysis of the signal, Fast Fourier Transformation (FFT) was calculated for 9–15 artifact-free 1 s samples taken from each CS presentation and transformed (z-scored). Peak power (Pmax) in the theta frequency band (4–12 Hz) and corresponding dominant frequency was calculated with Sudsa22.2S2 script. Pmax values obtained during each CS were afterwards normalized and compared between experimental groups. Theta synchronization between mPFC-vHPC during tone presentations (CS1-CS5) was determined by calculating signal cross-correlation via the Spike-2 software of low-pass (16 Hz) filtered waveforms. The level of the second positive peak in the cross-correlogram, corresponding to the theta frequency peak, was quantified after alignment to the maximal positive peak, averaged across individual animals and statistically assessed.

### Statistical analyses

Data were analyzed with the Prism 9 (GraphPad Sowftware Inc.) and R (version 3.3.3) software. Sample size was predetermined on the basis of published studies, experimental pilots and in-house expertize. Data are shown as mean ± SEM and, when informative, with individual values for each animal. Data distribution was tested for normality and successively analyzed with appropriate parametric or non-parametric statistical tests. Where applicable, multiple comparisons, following significant ANOVAs, were further analyzed using Bonferroni post hoc tests. Significance levels were set at p values less than 0.05.

## Results

### Anatomical and functional profiling of mGlu_5_ in SST+ neurons

To evaluate the functional implications of mGlu_5_ in SST+ neurons, we studied at first their co-expression in neurons from a number of brain areas that were selected based on previous in situ hybridization and immunocytochemical studies and showed large macroscopic overlap [15, 16, 47–50]. Consistent with previous studies, immunoreactivity for mGlu_5_ was widespread in the telencephalon, conversely SST+ neurons were observed primarily in the hypothalamus, LS and CeA, whereas they were scattered in the neocortex, HPC, basal amygdala and striatum (Fig. 1a, b). Because of the intense mGlu_5_ immunostaining in the neuropil and the SST labeling being mostly restricted to the Golgi apparatus and endoplasmic reticulum in neuronal somata, the study of their co-localization required high resolution confocal and electron microscopy (Fig. 1c–e and Suppl. Fig. 1). In cortical INs, we observed a 55-85% co-localization between mGlu_5_ and SST, depending on the area (Fig. 1c and Suppl. Fig. 1e; Suppl. Table 2). Different patterns of mGlu_5_ somatic staining were observed ranging from a plasma membrane-restricted immunoreactive signal to a dense cytoplasmic labeling (Fig. 1c, d and Suppl. Fig. 1a, e), suggesting a complex and cell-dependent trafficking [51, 52]. In the analyzed subcortical brain areas, namely the LS and CeA, we unexpectedly did not detect mGlu_5_ labeling in SST+ neurons (Fig. 1e; Suppl. Fig. 1 and Suppl. Table 2). In the CeA, mGlu_5_ immunoreactivity appeared to be primarily associated with PKCδ -expressing neurons (Suppl. Fig. 1c, d).

We next sought to investigate the intrinsic action of mGlu_5_ activation in SST+ INs in the hippocampus and neocortex using whole-cell patch-clamp recording. To identify SST+ INs in acute slices, we took advantage of SST^Cre^::Ai14 transgenic mice that express the reporter tdTomato in these neurons. First, hyperpolarizing and depolarizing current steps (1 s) were applied to assess passive and active membrane properties. After recording, biocytin-filled cells were morphologically reconstructed. SST+ INs exhibited diverse axonal arborization patterns, including bistratified and oriens-lacunosum moleculare (O-LM) projections. These INs also showed distinct firing properties from PNs (Fig. 1f, i, l; Suppl. Fig. 2g, i). In the CA1 field of the HPC, most SST+ INs exhibited low-threshold regular spiking properties (Fig. 1l) although we also recorded SST+ INs with fast spiking, adapting and stuttering firing patterns (Fig. 1i; Suppl. Fig. 2g), in line with the known heterogeneity of these INs [32, 35]. Similar diversity in firing properties was observed for the SST+ INs recorded in the temporal association cortex (Suppl. Fig. 2i).

**Fig. 2:**
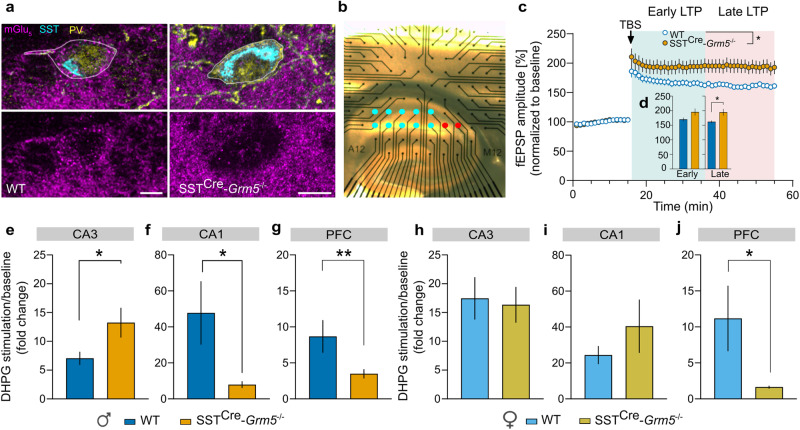
mGlu_5_ in SST+ neurons modulate network activity in a sex- and region-specific manner. **a** Example images of mGlu_5_ (magenta), SST (turquoise), and PV (yellow) immunoreactivity in O-LM INs from sections of a WT (left panels, scale bar 5 μm, thickness of the z-stack 2.21 μm) and a SST^Cre^-*Grm5*^−/−^ mouse brain (right panels, scale bar 5 μm, thickness of the z-stack: 3.45 μm). The absence of mGlu_5_ labeling along the plasma membrane (outlined in white in the merged upper panels) of SST^Cre^-*Grm5*^−/−^ O-LM INs confirms the recombination and, in turn deletion of *Grm5* from SST+ neurons. **b** Image depicting the MEA stimulating and recording electrodes position within a hippocampal mouse slice. Red circles represent stimulating electrodes and blue ones recording electrodes. **c** Upon TBS, slices from SST^Cre^-*Grm5*^−/−^ mice displayed increased LTP (fEPSP amplitude in %) compared to WT mice. Two-way ANOVA, Genotype *F*_1,73_ = 5.317, *p < 0.05. **d** The increased LTP in SST^Cre^-*Grm5*^−/−^ compared to WT mice was more prominent in the late LTP phase (Early LTP: Mann–Whitney *U* = 514, *p* > 0.05; Late LTP: Mann–Whitney *U* = 558, *p < 0.05). **e** Fold change frequency ratios upon DHPG stimulation in the CA3 region of the HPC were larger in SST^Cre^-*Grm5*^−/−^ compared to WT male mice. Mann–Whitney *U* = 20884, *p < 0.05. **f** Fold change frequency ratios upon DHPG stimulation in the CA1 region of the HPC were reduced in SST^Cre^-*Grm5*^−/−^ compared to WT male mice. Mann–Whitney *U* = 24475, *p < 0.05. **g** Fold change frequency ratios upon DHPG stimulation in the mPFC were reduced in SST^Cre^-*Grm5*^−/−^ compared to WT male mice. Mann-Whitney U = 49767, **p < 0.01. **h**, **i** Fold change frequency ratios upon DHPG stimulation in the CA3 (Mann–Whitney *U* = 28391, *p* > 0.05) (**h**) and CA1 (Mann–Whitney *U* = 11080, *p* > 0.05), **i** region of the HPC was similar in SST^Cre^-*Grm5*^−/−^ and WT female mice. **j** Fold change frequency ratios upon DHPG stimulation in the mPFC were reduced in SST^Cre^-*Grm5*^−/−^ compared to WT female mice. (Mann–Whitney *U* = 56486, *p < 0.05). Data are shown as mean ± SEM. *p < 0.05, **p < 0.01.

The bath application of the group I mGlu agonist DHPG (10 µM) increased the firing frequency and led to a pronounced membrane depolarization in the majority of the recorded SST+ INs (*n* = 31 out of 41; 76%) in HPC and neocortex as well as in essentially all CA1 PNs (*n* = 14; Fig. 1g, h, Suppl. Fig. 2a–c). The mean membrane depolarization change was 9.86 ± 2.43 mV and 8.75 ± 1.42 mV in CA1 PNs (*n* = 14/14) and SST+ INs (*n* = 16/18), respectively. The selective mGlu_5_ NAM MTEP (10 µM) effectively antagonized the action of DHPG on the CA1 PNs (*n* = 5, *p* < 0.05) as well as on the majority of CA1 SST+ INs (*n* = 6, *p* < 0.05) (Fig. 1h, k). In the presence of the mGlu_1_ NAM JNJ16259685 (500 nM), DHPG was still able to induce a significant depolarization in CA1 (*n* = 6) and neocortical (*n* = 5) SST+ INs (Fig. 1l–n; Suppl., Fig. 2i–k).

These findings demonstrate functional expression of mGlu_5_ in a large proportion of cortical SST+ INs, belonging to different subtypes. The activation of mGlu_5_ in these INs led to membrane depolarization and to an increase in firing frequency. Despite we did not detect a co-localization between mGlu_5_ and SST in neurons of the LS and CeA, we cannot rule out, only based on our immunocytochemical experiments, that these neurons, or some of them, may actually contain the receptor, though at levels below our threshold of detection at the soma. Future experiments are warranted to resolve this issue using other methodological approaches.

### mGlu_5_ in SST+ neurons regulate synaptic plasticity and local network activity

Next, we generated a mouse line with a selective deletion of mGlu_5_ in SST+ neurons via Cre-mediated recombination (Suppl. Fig. 3a) to explore the functional relevance of mGlu_5_ in these neurons. RNAscope, immunofluorescence staining and biochemical assays were used to evaluate cell and tissue-specific removal of mGlu_5_ expression. In mice with *Grm5* conditional KO (cKO) in SST+ neurons (SST^Cre^-*Grm*5^−/−^) no mRNA or protein fluorescent labeling for mGlu_5_ was detected in cortical SST+ INs (Fig. 2a; Suppl. Fig. 3b), that was consistent with a small reduction in overall mGlu_5_ protein levels as measured by western blot analysis (Suppl. Fig. 3c–f). Furthermore, in hippocampal slices of SST^Cre^-*Grm*5^−/−^ mice, bath application of DHPG (10 µM) failed to increase the membrane potential of SST+ INs (*n* = 6) in the presence of JNJ16259685 (Suppl. Fig. 4).

Since mGlu_5_ plays an important role in synaptic plasticity [10, 11], we first investigated the functional influence of *Grm5*-cKO in SST+ neurons (SST^Cre^-*Grm*5^−/−^) on the induction and maintenance of LTP in the hippocampal Schaffer collateral-CA1 pathway using MEA recordings (Fig. 2b). Previous work showed that germline gene-targeted deletion or selective antagonism of mGlu_5_ markedly reduced LTP at this synapse [53–56]. In slices from both SST^Cre^-*Grm*5^−/−^ and littermate control animals, TBS of the Schaffer collaterals consistently induced a long-lasting increase of fEPSP amplitudes (Fig. 2c). However, in SST^Cre^-*Grm5*^−/−^ mice LTP was further facilitated when compared to littermate controls (Fig. 2c, d).

Ex vivo recordings of hippocampal network activity yielded subregion- and sex-specific differences in frequency ratios upon stimulation with DHPG (Fig. 2e–j; Suppl. Fig. 5a–g). In male, but not female SST^Cre^-*Grm*5^−/−^ mice, we observed higher frequency ratios compared to baseline in CA3 and lower frequency ratios in CA1, as compared to littermate controls (Fig. 2e, f, h, i). Conversely, in the mPFC both male and female SST^Cre^-*Grm*5^−/−^ mice displayed decreased frequency ratios compared to baseline upon DHPG application (Fig. 2g, j). However, these changes in local network activity were not common for other neocortical structures such as the dorsal anterior cingulate cortex (dACC; Suppl. Fig. 5h, i), suggesting that mGlu_5_ in SST+ INs can increase or decrease local network activity in a region-specific manner across different cortical structures.

To explore the relative contribution of mGlu_1_ and mGlu_5_, given their complex pattern of co-expression in the HPC [16, 57], we performed MEA excitability recordings in slices from control mice, in the presence of the mGlu_1_ and mGlu_5_ NAMs JNJ16259685 and MTEP, respectively. In the CA1 subfield, MTEP produced a much stronger reduction of DHPG-mediated activity than JNJ16259685 (Suppl. Fig. 5j). On the other hand, in the CA3 DHPG-mediated activity was more effectively lowered in the presence of JNJ16259685 as compared to MTEP (Suppl. Fig. 5k). The combination of JNJ16259685 and MTEP fully suppressed DHPG-stimulated activity (Suppl. Fig. 5j, k). These findings suggest that the effects onto local network activity are preferentially mediated by mGlu_5_ in the CA1 and by mGlu_1_ in the CA3.

Altogether, these results show that the loss of mGlu_5_ in SST+ INs facilitates synaptic plasticity in CA1 PNs, most likely through the reduction of dendritic inhibition by these INs, and induces area- and sex-specific changes of local network activity.

### mGlu_5_ in SST+ neurons modulate social and anxiety-like behavior in a sex-specific manner

Previous studies have shown that germline *Grm5* deletion or systemic application of mGlu_5_ antagonists change the expression of several emotional behaviors including social interaction, anxiety-like behavior and fear responses [41, 46, 58]. Selective deletion of *Grm5* in forebrain GABAergic neurons, but not in glutamatergic PNs [22], to some extent mimics these changes on emotional states [24]. The specific ablation of mGlu_5_ in PV+ neurons did not seem to importantly contribute to deficits in anxiety-like behavior, and the influence on sociability was attributed to cognitive deficits [25].

**Fig. 3:**
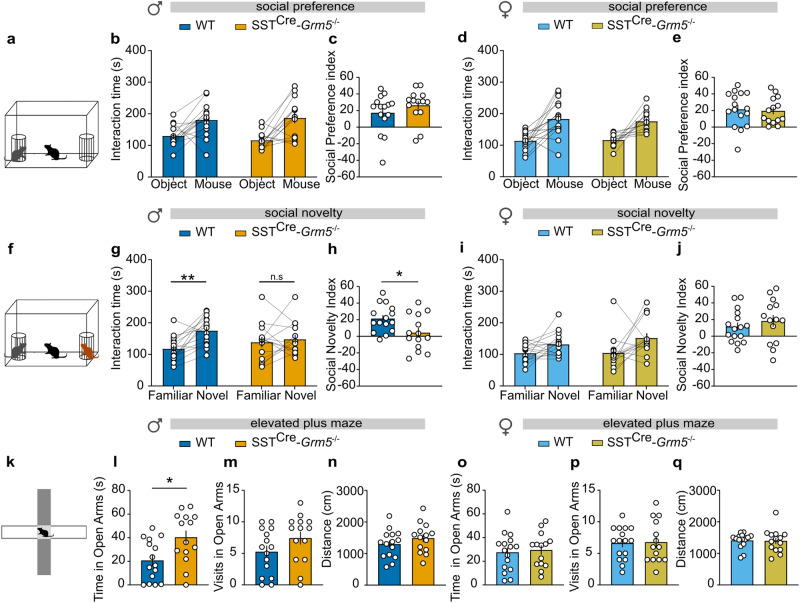
mGlu_5_ in SST+ neurons modulate social behaviors and anxiety in male mice. **a** Schematic of the social preference test. **b**, **d** No significant differences between WT and SST^Cre^-*Grm5*^−/−^ animals were observed in social interaction time in males (**b**) (two-way ANOVA, *F*_1,27_ = 0.69, *p* > 0.05) or females (**d**) (two-way ANOVA, *F*_1,28_ = 0.20, *p* > 0.05). **c**, **e** Similarly, no differences were observed in the social preference index in either male (unpaired *t* test, *t*_27_ = 1.1, *p* > 0.05) or female mice (unpaired *t* test, *t*_28_ = 0.25, *p* > 0.05). **f** Schematic of the social novelty test. **g**, **h** Male SST^Cre^-*Grm5*^−/−^ did not show social novelty preference as compared to WT mice, both measured as interaction time (**g**) (two-way ANOVA, Interaction *F*_1,27_ = 4.75, *p < 0.05; Bonferroni multiple comparisons test: **p < 0.01, *p* > 0.05) and social novelty index (**h**) (unpaired *t* test, *t*_27_ = 2.39, *p < 0.05). **i**, **j** Both WT and SST^Cre^-*Grm5*^−/−^ female mice displayed similar social novelty preference (**i**) (two-way ANOVA, *F*_1,28_ = 0.63, *p* > 0.05), and social novelty index (**j**) (unpaired *t* test, *t*_28_ = 0.80, *p* > 0.05). **k** Schematic of the elevated plus maze test. **l**–**n** While WT and SST^Cre^-*Grm5*^−/−^ animals traveled a similar distance and visited the open arms of the elevated plus maze to a similar extent (**m**) (unpaired *t* test, *t*_27_ = 1.13, *p* > 0.05; **n** Mann–Whitney, *U* = 68, *p* > 0.05), SST^Cre^-*Grm5*^−/−^ mice spent more time in the open arms of the maze as compared to WT (**l**) (unpaired *t* test, *t*_27_ = 2.73, *p < 0.05). **o**–**q** Both WT and SST^Cre^-*Grm5*^−/−^ female mice showed similar performances during the elevated plus maze test (**o**) (unpaired *t* test, *t*_28_ = 0.09, *p* > 0.05; **p** Mann–Whitney, *U* = 110, *p* > 0.05; **q** unpaired *t* test, *t*_28_ = 0.35, *p* > 0.05). Data are shown as mean ± SEM, and individual data points are also provided. *p < 0.05. **p < 0.01.

SST^Cre^-*Grm5*^−/−^ mice showed no overt phenotypic alterations in development or locomotion (Suppl. Fig. 6a, b). We then examined how the loss of mGlu_5_ in SST+ neurons influenced a variety of behaviors in the cognitive, social and negative valence domains (Suppl. Fig. 6c). Male as well as female SST^Cre^-*Grm5*^−/−^ mice showed similar social preference to that of littermate controls in the classical three-chamber social interaction test (Fig. 3a–e). When further tested for social novelty, only male SST^Cre^-*Grm5*^−/−^ mice displayed reduced preference for the novel interactor (Fig. 3f–h). The lack of social novelty differences between genotypes in female mice (Fig. 3i, j), however, might have been affected by a lower sensitivity of the test in this sex under our experimental conditions, as evidenced by a less pronounced preference. The deletion of *Grm5* in SST+ neurons did not result in any detectable deficit in novel object exploration and in the marble burying test (Suppl. Fig. 6d, e). When tested for measures of anxiety-like behavior, SST^Cre^-*Grm5*^−/−^ male, but not female, mice spent more time in the open arms of the elevated plus maze test as compared to control animals (Fig. 3l). This apparent anxiolytic activity in male mice did not arise from a novelty-induced hyper-locomotion (Fig. 3m, n; Suppl. Fig. 6b).

Altogether these data suggest sexual dimorphism in the contribution of mGlu_5_ to the regulation of social and anxiety-like behavior by SST+ neurons.

### mGlu_5_ in SST+ neurons modulate fear expression in an estrous-dependent manner

**Fig. 4:**
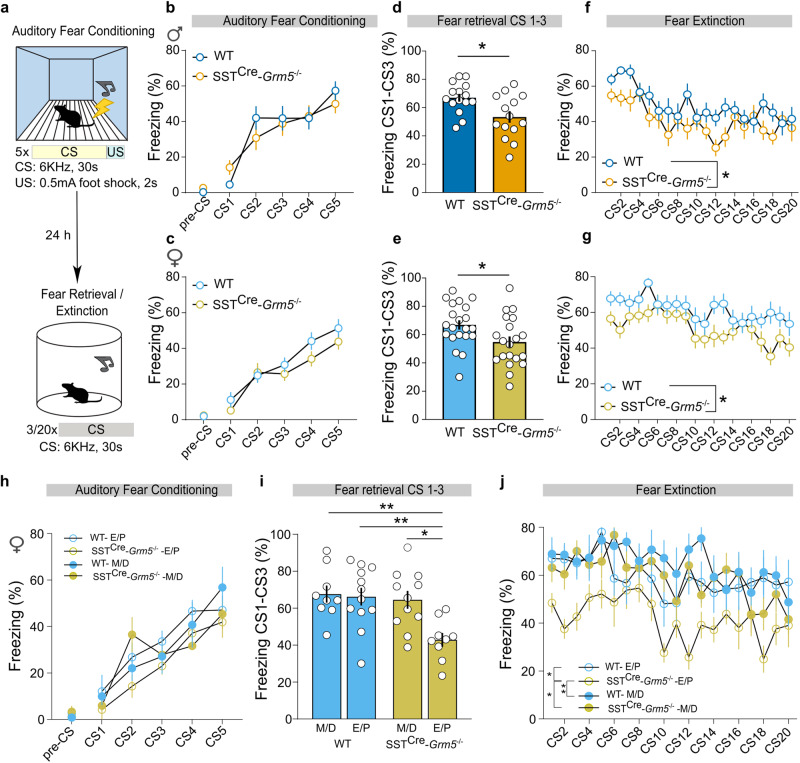
mGlu_5_ in SST+ neurons modulate fear expression. **a** Experimental protocol of the auditory fear conditioning, retrieval, and extinction paradigms. **b**, **c** Littermate controls (WT) and SST^Cre^-*Grm5*^−/−^ mice displayed similar acquisition of the conditioned response during the fear conditioning session. **b** Males: two-way ANOVA, interaction *F*_5,135_ = 1.35, *p* > 0.05; CS *F*_5,135_ = 40.87, *p* < 0.0001; Genotype *F*_1,27_ = 0.08, *p* > 0.05; **c** Females: two-way ANOVA, Interaction *F*_5,195_ = 0.89, *p* > 0.05; CS *F*_5,195_ = 51.96, *p* < 0.0001; Genotype *F*_1,39_ = 1.52, *p* > 0.05). **d**, **e** SST^Cre^-*Grm5*^−/−^ mice, both males and females, displayed lower freezing levels during fear retrieval (first three CS presentations) as compared to WT mice. **d** Males: unpaired *t* test, *t*_27_ = 2.76, *p < 0.05; **e** Females: unpaired *t* test, *t*_39_ = 2.35, *p < 0.05). **f**, **g** SST^Cre^-*Grm5*^−/−^ mice also showed a slightly enhanced fear extinction with respect to WT mice. **f** Males: two-way ANOVA, Interaction *F*_19,513_ = 0.99, *p* > 0.05, Genotype *F*_1,27_ = 5.024, *p < 0.05; **g** Females: two-way ANOVA, Interaction *F*_19,741_ = 1.08, *p* > 0.05, Genotype *F*_1,39_ = 5.16, *p < 0.05). **h** WT and SST^Cre^-*Grm5*^−/−^ female mice at different estrous cycle stages, estrous/proestrous (E/P) and metestrous/diestrous (M/D), displayed similar acquisition of the conditioned response during the fear conditioning session. **i**, **j** SST^Cre^-*Grm5*^−/−^ female mice during the E/P phase displayed lower freezing levels during fear retrieval (**i**) and extinction (**j**) as compared to SST^Cre^-*Grm5*^−/−^ female mice during the M/D phase and WT females irrespective of their estrous cycle. **i** Two-way ANOVA, Interaction *F*_1,37_ = 4.663, *p* < 0.05; Estrous *F*_1,37_ = 7.995 *p* < 0.005; Genotype *F*_1,37_ = 6.076, *p* < 0.05. Bonferroni multiple comparisons test: *p < 0.05, **p < 0.01. **j** Two-way ANOVA, Interaction *F*_57,703_ = 1.073, *p* > 0.05; CS *F*_19,703_ = 4.209, *p* < 0.0001; Group *F*_3,37_ = 5.006, *p* = 0.005. Bonferroni multiple comparisons test: *p < 0.05, **p < 0.01. Data are shown as mean ± SEM. Individual data points are provided for fear retrieval data. *p < 0.05, **p < 0.01.

To further explore whether the loss of mGlu_5_ in SST+ neurons had additional effects on constructs of negative affect, we next used a Pavlovian fear conditioning paradigm (Fig. 4a). SST^Cre^-*Grm5*^−/−^ mice showed similar fear-learning responses in comparison to their respective littermate controls (Fig. 4b, c). In contrast, both male and female SST^Cre^-*Grm5*^−/−^ mice exhibited reduced freezing when subjected to fear retrieval in a novel context (Fig. 4d, e). The acquisition of fear extinction, which is generally considered a proxy of inhibitory learning [59–62], was also slightly facilitated by the depletion of mGlu_5_ in SST+ neurons. However, similar freezing levels were reached by both SST^Cre^-*Grm5*^−/−^ and control mice at the end of the extinction training (Fig. 4f, g).

In order to identify which traits better discriminate one genotype from another, and to reveal which behavioral domain is mostly affected by the loss of mGlu_5_ in SST+ neurons, we performed a random forest (RF) classification analysis with a leave-one-out cross-validation of all behavioral responses measured in SST^Cre^-*Grm*5^−/−^ and control mice (Suppl. Fig. 7a–d). The weight of each response to accurately differentiate genotypes was given by the Gini index, with the most discriminative ones being fear retrieval and anxiety-like behavior (Suppl. Fig. 7a). Interestingly, these two phenotypes, measured as time spent in the open arms of the EPM and freezing during fear retrieval, correlated only in WT male mice (Suppl. Fig. 7e–h). The general RF analysis, including all animals regardless of sex, led to a genotype prediction accuracy of 61%. Conversely, individual RF classifications taking into account the two sexes led to substantial differences in prediction errors. A RF classifier for male mice led to an accuracy close to 80% (24/30 mice correctly predicted), whereas a classifier for female mice remained at chance level (13/30 mice correctly predicted).

We next explored whether the lower prediction power of the behavioral classifiers in female mice would reflect potential estrous-dependent contributions in the negative valence domain (Fig. 4h–j). Upon stratification of the estrous stage following fear extinction, we observed that the reduced fear retrieval and facilitated extinction learning by the depletion of mGlu_5_ in SST+ neurons was only apparent in SST^Cre^-*Grm*5^−/−^ female mice in estrous-proestrus (Fig. 4i, j). Taken together our findings suggest that the lack of mGlu_5_ in SST+ neurons primarily affects the negative valence system. While this is more clearly observed in male mice, in female mice it appears to be estrous stage dependent.

### Generation of hippocampal theta rhythm during fear retrieval depends on mGlu_5_ in SST+ neurons

**Fig. 5:**
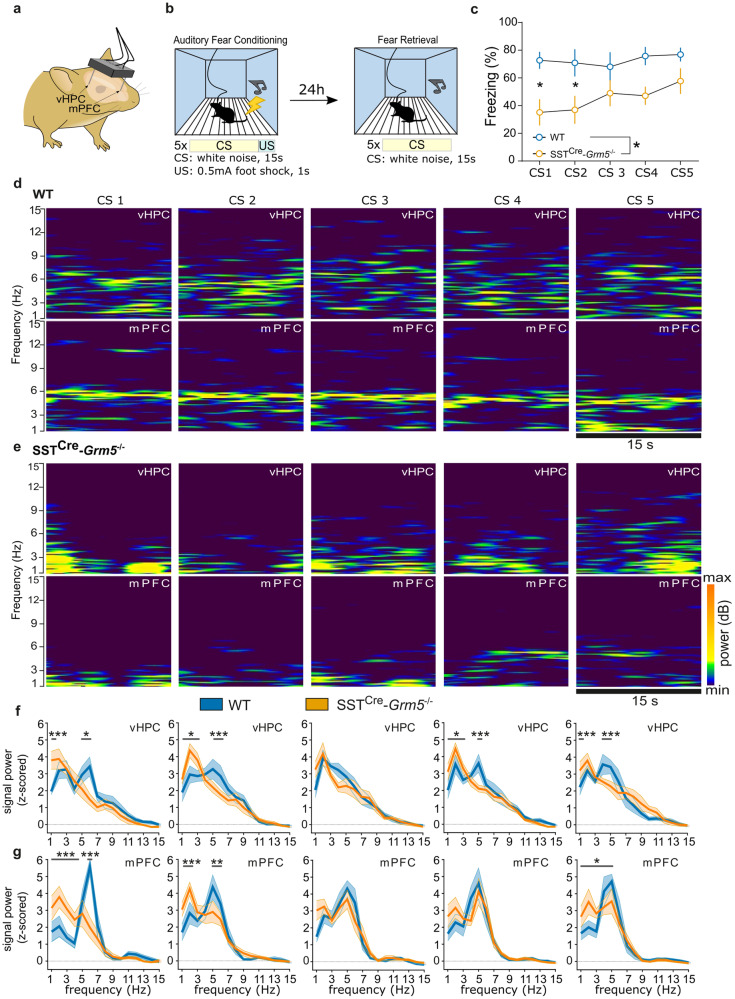
Ablation of mGlu_5_ in SST + INs impairs theta rhythm generation in the mPFC and vHPC during fear retrieval. **a** Schematic of the experimental approach used for freely moving mPFC and vHPC LFP electrophysiological recordings. **b** Experimental setup and protocol of the auditory fear conditioning and auditory/contextual retrieval paradigm. **c** SST^Cre^-*Grm*5^−/−^ mice showed lower freezing levels as compared to WT mice during fear retrieval (two-way ANOVA, Genotype *F*_1,11_ = 6.45, *p < 0.05) that were more prominent during the initial CS presentations (CS1-CS2; *p < 0.05). **d**, **e** Examples of LFP expressed as sonograms, recorded from the vHPC and mPFC during the CS1-CS5 presentations in a fear retrieval session in WT (**d**) and SST^Cre^-*Grm*5^−/−^ mice (**e**). **f**, **g** Power spectra (z-scored) of LFP signals recorded from the vHPC or mPFC. The analysis of the entire frequency range of the power spectra (1–75 Hz) of the vHPC signal (**f**) during the different CS presentations revealed differences between WT and SST^Cre^-*Grm*5^−/−^ mice in the delta and theta range. CS1: two-way ANOVA, Interaction *F*_74, 825_ = 2.60, ***p < 0.001; CS2: two-way ANOVA, Interaction *F*_74, 825_ = 2.19, ***p < 0.001; CS3: two-way ANOVA, Interaction *F*_74,749_ = 0.54, *p* > 0.05; CS4: two-way ANOVA, Interaction *F*_74,825_ = 2.13, ***p < 0.001; CS5: two-way ANOVA, Interaction *F*_74,825_ = 1.56, ***p < 0.001. Significance in the graphs depicts *post hoc* Bonferroni multiple comparisons test: *p < 0.05, ***p < 0.001. The analysis of the entire frequency range of the power spectra (1–75 Hz) of the mPFC signal (**g**) during the different CS presentations revealed differences between WT and SSTCre-Grm5−/− mice in the delta and theta range. CS1: two-way ANOVA, Interaction F_74, 814_ = 4.86, ***p < 0.001; CS2: two-way ANOVA, Interaction *F*_74,814_ = 2.09, **p < 0.01; CS3: two-way ANOVA, Interaction *F*_74,814_ = 1.19, *p* > 0.05; CS4: two-way ANOVA, Interaction *F*_74, 814_ = 0.86, *p* > 0.05; CS5: two-way ANOVA, Interaction *F*_74,814_ = 1.52, *p < 0.05. Significance in the graphs depicts post hoc Bonferroni multiple comparisons test: *p < 0.05, **p < 0.01, ***p < 0.001. Data are shown as mean ± SEM.

Negative emotional valence induced by anxiety or fear enhances the generation of theta frequency in HPC [63] and mPFC [40], as well as their synchronized activity [40]. Since SST+ INs facilitate HPC-mPFC synchrony [38], we sought to characterize the local neural oscillatory activity in the vHPC and mPFC in mice lacking mGlu_5_ in SST+ neurons. SST^Cre^-*Grm5*^−/−^ and littermate control male mice were implanted with LFP recording electrodes in the vHPC and mPFC (Fig. 5a; Suppl. Fig. 8a, b) and tested for the most discriminative behavioral trait between the two genotypes (Suppl. Fig. 7a), namely fear retrieval following a Pavlovian fear conditioning paradigm. However, in this paradigm fear retrieval was carried out in the same context where the conditioning was performed (Fig. 5b) in order to induce stronger theta activity during CS presentations. Consistent with our previous experimental protocol, SST^Cre^-*Grm5*^−/−^ mice froze less than controls during the initial CS presentations (Fig. 5c). This behavioral observation was accompanied with substantial changes in the signal power spectra (FFT) between SST^Cre^-*Grm5*^−/−^ and control mice (Fig. 5d, e). This dissimilarity was particularly evident during the first two CS presentations as a pronounced reduction of spectral content (peak power) in the theta frequency band (4–12 Hz) in SST^Cre^-*Grm5*^−/−^ mice, both in the vHPC and in the mPFC (Fig. 5f, g; Suppl. Fig. 9a, b). During the last 3 CS presentations, theta activity gradually resumed in the *Grm5* cKO animals, suggesting that lower theta power during CS presentations did not arise from a general inability of SST^Cre^-*Grm5*^−/−^ mice to generate theta rhythms (Fig. 5f, g; Suppl. Fig. 8c). The dominant frequency in the theta frequency band during the CS presentations was at 5–6 Hz in both WT and SST^Cre^-*Grm5*^−/−^ animals (Suppl. Fig. 9c), consistent with type 2 theta oscillations (4–8 Hz) known to be dominant during periods of fear-induced immobility [63–65]. The maximal peak power (Pmax) in the mPFC showed a positive correlation with the percent of freezing measured during the 5 CS presentations (Suppl. Fig. 9d).

**Fig. 6:**
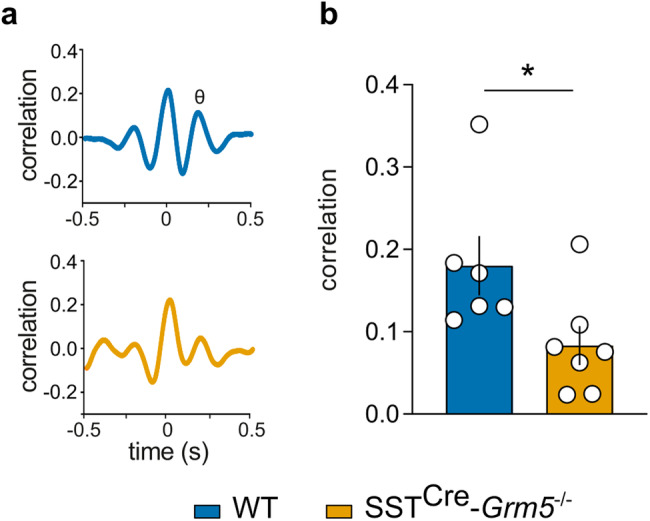
Ablation of mGlu_5_ receptors in SST + INs disrupts network activity synchronization between the mPFC and vHPC. **a** Theta phase correlation level and **b** average cross-correlation values for the second positive peak of mPFC/vHPC activity during freezing expression within all CS presentations. Unpaired *t* test, *t*_11_ = 2.37, *p < 0.05. Data are shown as mean ± SEM and individual values.

Finally, we assessed the LFP signal synchronization (theta phase correlation level) between the mPFC and vHPC in relation to negative valence processing in SST^Cre^-*Grm*5^−/−^mice. Cross-correlation analysis of network activity upon fear retrieval revealed a significant reduction of theta synchronization between the two brain areas during freezing episodes in comparison to WT animals (Fig. 6a, b).

Altogether these results suggest that mGlu_5_ in SST+ neurons are crucial for the entraining of synchronous theta oscillatory activity in mPFC and vHPC during negative valence processing.

## Discussion

Understanding the mechanisms by which cell- and circuit-specific alterations in discrete neurotransmitter receptor function influence the balance between cortical GABAergic and glutamatergic systems is critical for elucidating the fundamental neurobiology underlying many psychiatric disorders.

Here we report that the loss of mGlu_5_ in SST+ neurons decreased anxiety-like behavior and freezing during the retrieval of fear memory involving a reduction in theta frequency oscillatory activity in the mPFC and vHPC. Our study provides a circuit and functional mechanism contributing to the alleviation of anxiety and affective symptoms exerted by mGlu_5_ NAMs in both preclinical and clinical studies [8, 66–69]. In particular, we established that the co-expression of mGlu_5_ and SST occurs primarily in cortical INs, though not all of them displayed functional responses to the group I mGlu orthosteric agonist DHPG or labeling for mGlu_5_. The latter ranged from 55-85% according to the cortical area, with the hippocampal CA1 and mPFC apparently having the higher rate of co-localization. We further provided first evidence that ex vivo network activity following stimulation with DHPG was subregion- and sex-specific, most likely reflecting differences in cytoarchitectonics, intrinsic connectivity, diversity of SST+ INs and expression of group I mGlus in distinct neuronal populations. In particular, the known discrete cellular distribution of mGlu_5_ and mGlu_1_ in the HPC [70] and distinct, or even opposing, effects of these receptors on the excitability of hippocampal CA3 neurons [71] are consistent with the differential DHPG-mediated activity in distinct hippocampal subfields. Future studies should address how cell- and sex-specific changes in mGlu_5_ expression and function contribute to these differences. In this respect, it is particularly important for the SST+ INs given their high transcriptomic [72], anatomic and electrophysiologic [32] variability.

In the HPC, the loss of mGlu_5_ in SST+ INs facilitated Schaffer collateral to CA1 PN synaptic plasticity. This is fully consistent with a recent finding describing a role for SST+ INs in disinhibition-assisted LTP in the mPFC to amygdala pathway [73]. Our data suggest that mGlu_5_ in cortical SST+ INs, that mostly form GABAergic synapses onto dendritic spines of PNs [74, 75], by facilitating the activation of these INs help to control dendritic spike generation and synaptic plasticity in principal cells [76].

Behaviorally, the genetic-mediated ablation of *Grm5* selectively in SST+ neurons induced several alterations in domains of social processing and negative valence that were mostly apparent in males. Our data are consistent with previous work showing that chronic inhibition of cortical SST+ INs, particularly in the mPFC, reduces anxiety-like behavior and overall emotionality under baseline conditions and after chronic stress in adult mice [77, 78]. Surprisingly, disinhibition of SST+ neurons through inactivation of the γ2 subunit of the GABA-A receptor in these neurons also elicited an anxiolytic-like phenotype [79] apparently at odds with our and other studies [80]. On the other hand, the ubiquitous expression of γ2-containing GABA-A receptors in SST+ neurons including the CEA and LS, unlike mGlu_5_, might contribute to explain this discrepancy. In addition, a recent study from Joffe and coworkers showed that restraint stress rapidly potentiates excitatory drive onto SST+ INs in the mPFC through an mGlu_5_-dependent LTP at BLA inputs, which shunted incoming information from other circuits [81]. This mechanism of network processing and gating of information in the mPFC involving mGlu_5_ promoted cognitive behavioral adaptations related to acute stress [81]. The authors of this study also generated SST^Cre^-*Grm5*^−/−^ mice to confirm that mGlu_5_ on SST+ INs mediates restraint stress-induced changes in mPFC physiology, and reported that the loss of mGlu_5_ in SST+ neurons prevented stress-induced behavioral adaptations, but did not change baseline anxiety-like behavior [81]. However, since in this study behavioral data from both male and female mice were pooled, group differences might have been overlooked. Variations in baseline stress, e.g. introduced by subjecting the mice to repeated trials as in our protocol, may offer an alternative explanation. On the other hand, it is worth mentioning that Joffe and coworkers similarly observed in SST^Cre^-*Grm5*^−/−^ mice a reduction in freezing upon fear recall after cued-fear conditioning [81].

Using a RF analysis of the individual performances in the behavioral tests, we found that fear retrieval was the most discriminating construct between WT and SST^Cre^-*Grm5*^−/−^ mice and that the lack of mGlu_5_ in SST+ neurons could be strongly predicted based on behavioral performances in male, but not in female mice. The estrous cycle in females is known to significantly affect a number of behavioral responses [82–85]. Estrogen receptors (ERs) were reported to interact with group I mGlus [86, 87] in female but not in male rodents [88], and this interaction produced sex-dependent responses in conflict-based tests of anxiety-like behavior [89, 90]. Our results indeed suggest that the loss of mGlu_5_ in SST+ neurons affect negative valence specifically during the estrous-proestrous phase, thus supporting a cooperative regulation between ERs and mGlu_5_ in SST+ neurons on fear expression. An interesting open question remains as to whether these interactions might have been responsible for the sex-differences we observed in both the intrinsic network excitability in vitro and in some of the behavioral tests.

A key finding of our study is that, in addition to altered local network activity, the lack of mGlu_5_ in SST+ neurons reduced theta oscillatory activity in the mPFC and vHPC during fear memory retrieval. The marked reduction in theta activity did not arise from a general inability to generate theta oscillations or a shift in the dominant frequency within the theta frequency range, but rather as a result of weakened theta power. Exposure to anxiogenic and threatening contexts triggers the emergence of theta oscillations and their synchronization in the vHPC and mPFC [40, 91–93]. INs play a well-established role in governing oscillatory states [39, 94]. SST+ INs are sharply tuned to theta oscillations [91, 95] and enable HPC-PFC synchrony [38]. Previous studies have shown an important contribution in particular of mPFC SST+ INs in the behavioral expression of fear, conceivably as a result of the disinhibition of mPFC output neurons [27] and consequently the coordination of long range synchrony and information flow between the vHPC and mPFC [38]. Consistent with these studies, we show that in SST^Cre^-*Grm5*^−/−^ mice theta synchronization between vHPC and mPFC is markedly decreased in fear retrieval. This suggests that mGlu_5_ on SST+ neurons regulate frequency-specific synchronization between regions and may provide a potential molecular substrate for understanding changes in functional connectivity associated with negative affective states [96]. Thus, mGlu_5_ could act as gatekeepers of SST + IN inhibitory control onto anxiety network hubs [97], thus indirectly controlling the excitation/inhibition balance underlying the anxiety state of the organism.

Despite the large body of evidence supporting a contribution of mGlu_5_ to the development of or susceptibility to affective and anxiety disorders [8, 66, 67, 69, 98, 99], little was known about the underlying cellular pathophysiological mechanisms. Our study provides a novel mechanistic insight that imbalances in emotional behavior homeostasis, mainly regarding the negative valence domain, can ensue from the long-term loss of mGlu_5_ activity in SST+ neurons. While in SST^Cre^-*Grm5*^−/−^ mice region- and sex-specific functional impairments were most likely dependent on specific subclasses of cortical INs, we at present cannot rule out the possible contribution of sparse subcortical neurons co-expressing SST and mGlu_5_ or of cortical INs in which co-expression could not be detected.

In conclusion, our study advocates for a distributed but discrete role of mGlu_5_ in regulating SST+ neuron excitability and brain oscillatory activity. We envisage that mGlu_5_ are crucial determinants in the control that SST+ INs exert over the excitation/inhibition balance across brain regions and neuronal circuits mediating negative emotional states in health and disease.

## Supplementary information

Supplemental Material

## Data Availability

The data supporting this study are available from the corresponding author upon reasonable request. Datasets of raw and filtered local field potentials (LFPs) during fear memory recall generated during this study are available at Mendeley Data: 10.17632/z8nx775tkp.1.

## Code availability

The code for analyzing the data is available from the corresponding author upon reasonable request.
