# Supplementary material for: Loss of mGlu5 receptors in somatostatin-expressing neurons alters negative emotional states
Source: Mol Psychiatry. 2024 Apr 4;29(9):2774–86. doi: 10.1038/s41380-024-02541-5 (PMC11420089; doi:10.1038/s41380-024-02541-5)
Supplement: Supplementary file 1 — Supplemental Material [file 41380_2024_2541_MOESM1_ESM.pdf]

SUPPLEMENTARY FIGURES

Loss of mGlu$_5$ receptors in somatostatin-expressing neurons alters negative emotional states

Arnau Ramos-Prats, PhD[1,¶]; Pawel Matulewicz, PhD[1]; Marie-Luise Edenhofer, PhD[2], Kai-Yi Wang, PhD[3]; Chia-Wei Yeh, PhD[3]; Ana Fajardo-Serrano, PhD[1]; Michaela Kress, MD[2], Kai K. Kummer, PhD[2]; Cheng-Chang Lien, MD, PhD[3] and Francesco Ferraguti, MD[1*]

[1]Institute of Pharmacology, Medical University of Innsbruck, Innsbruck, Austria

[2]Institute of Physiology, Medical University of Innsbruck, Innsbruck, Austria

[3]Institute of Neuroscience, National Yang Ming Chiao Tung University, Taipei, Taiwan

[¶] Present address: Friedrich Miescher Institute for Biomedical Research, Basel, Switzerland

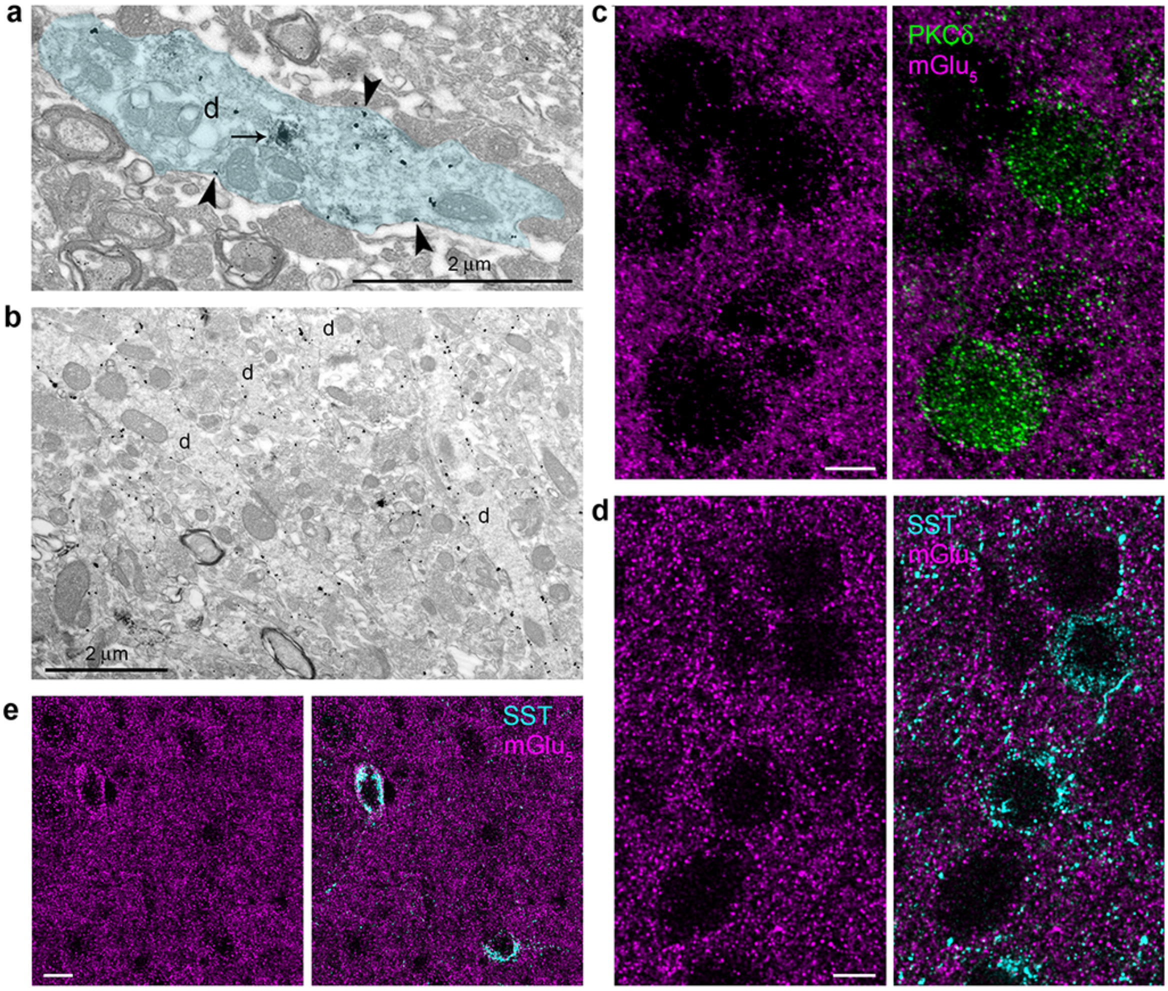

Suppl. Figure 1. Anatomical characterization of mGlu5 expression in SST+ neurons.

(a) Electron micrograph of a CA1 oriens/alveus IN dendrite labeled for SST+ (visible as DAB electron-dense deposit in the endoplasmic reticulum, indicated by the arrow) and showing immunometal particles distinguishing $mGlu_5$ both in the cytoplasm and at the plasma membrane (arrowheads). Scale bar: 2 µm. **(b)** Electron micrograph of CA1 pyramidal cell dendrites in the stratum radiatum, that unlike SST+ INs display $mGlu_5$ immunolabeling (gold-silver particles) almost exclusively associated to the plasma membrane. Scale bar: 2 µm. **(c)** Representative confocal image stack of $mGlu_5$ (in magenta) and PKCδ (green) co-expression in neurons of the mouse lateral CeA. Left panel, $mGlu_5$ immunolabeling; right panel, merged image. **(d)** Representative confocal image stack of $mGlu_5$ (magenta) and SST (turquoise) in neurons of the mouse lateral CeA. Left panel, $mGlu_5$ immunolabeling; right panel, merged

image. SST labeled neurons lack $mGlu_5$ immunostaining on the somatic plasma membrane, which on the other hand can be observed in SST immunonegative cells. SST immunolabeling can also be observed in the neuropil in axons and presynaptic terminals. **(e)** Representative confocal image stack of $mGlu_5$ (magenta) and SST (turquoise) co-expression in neocortical INs displaying high intracytoplasmic $mGlu_5$ immunofluorescence signal. Thickness of the confocal z-stacks: **(c)** 1.54 µm; **(d)** 1.36 µm, **(e)** 6.02 µm. Scale bars: **(c-d)** 5 µm, **(e)** 8 µm.

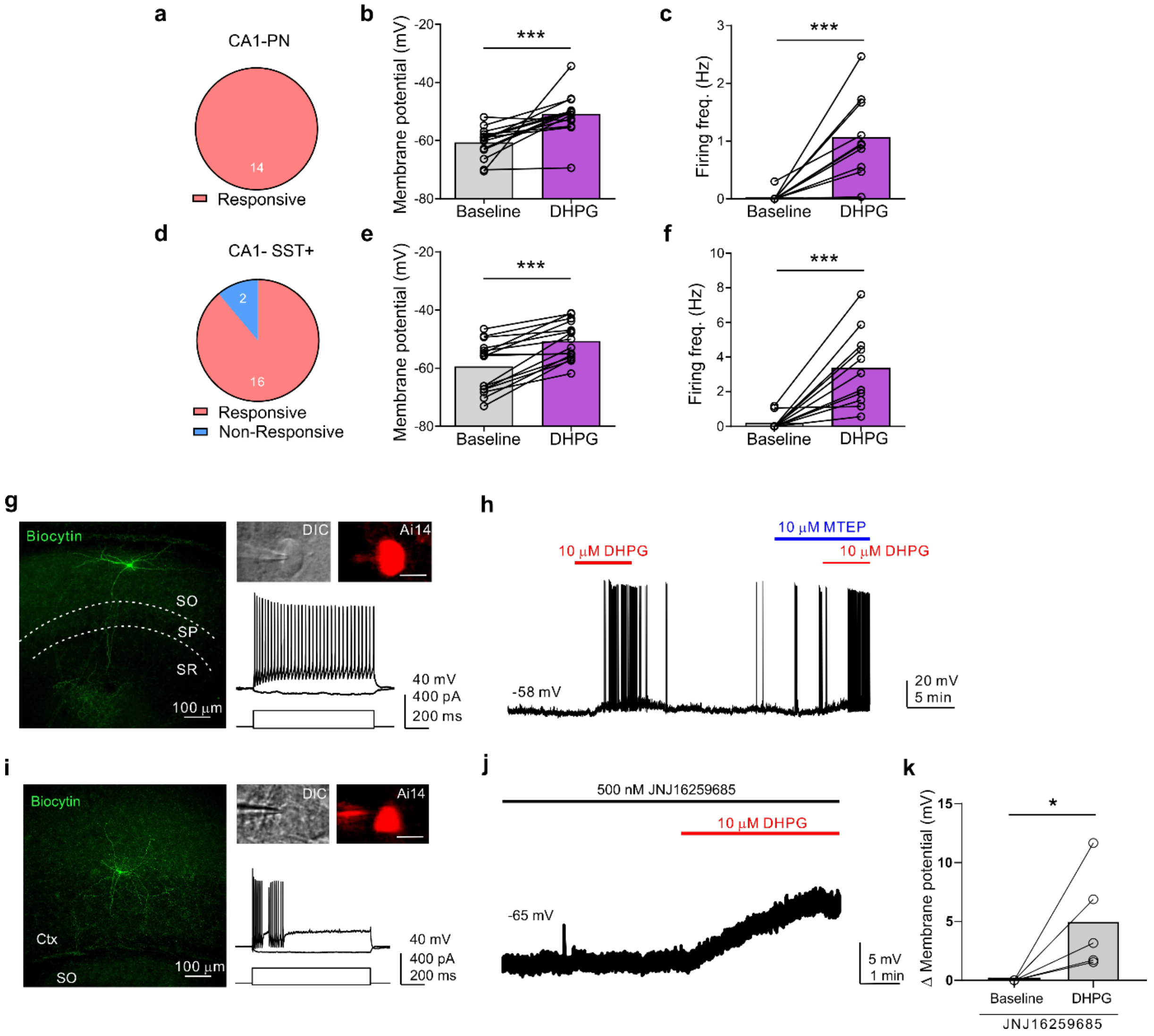

Suppl. Figure 2. mGlu$_5$ modulate excitability of PN and SST+ neurons.

(a) All recorded CA1 PNs responded to DHPG infusion. **(b-c)** DHPG infusion increased membrane potential (14/14) **(b)** (Wilcoxon matched-pairs rank test, W=101) and firing frequency (11/14) in PNs **(c)** (Wilcoxon matched-pairs rank test, W=66)**.** **(d)** In CA1, 16/18 recorded SST+ INs responded to DHPG infusion. **(e-f)** DHPG infusion increased membrane potential (16/18) **(e)** (Wilcoxon matched-pairs rank test, W=136) and firing frequency (11/18) in CA1 SST+ INs **(f)** (Wilcoxon matched-pairs rank test, W=66). Plots in **b-c, e-f** represent mean and individual values. ***p<0.001. **(g)** Example of a Biocytin-filled CA1 SST+ IN with its respective DIC image and membrane responses to current injections. **(h)** Example trace displaying membrane depolarization upon DHPG infusion and lack of blockage upon MTEP

pre-treatment in an O-LM cell. **(i)** Representative example of a biocytin-filled cortical SST+ IN with its respective DIC image and membrane responses to current injections. **(j)** Example trace displaying membrane depolarization upon DHPG application in the presence of 500 nM of the $mGlu_1$ antagonist JNJ16259685. **(k)** Quantification of DHPG-mediated membrane potential change in cortical SST+ INs in the presence of JNJ16259685 (n=5). Wilcoxon matched-pairs rank test, W=15, *p<0.03. Data are shown as mean and individual data points. *p<0.05. ***p<0.001.

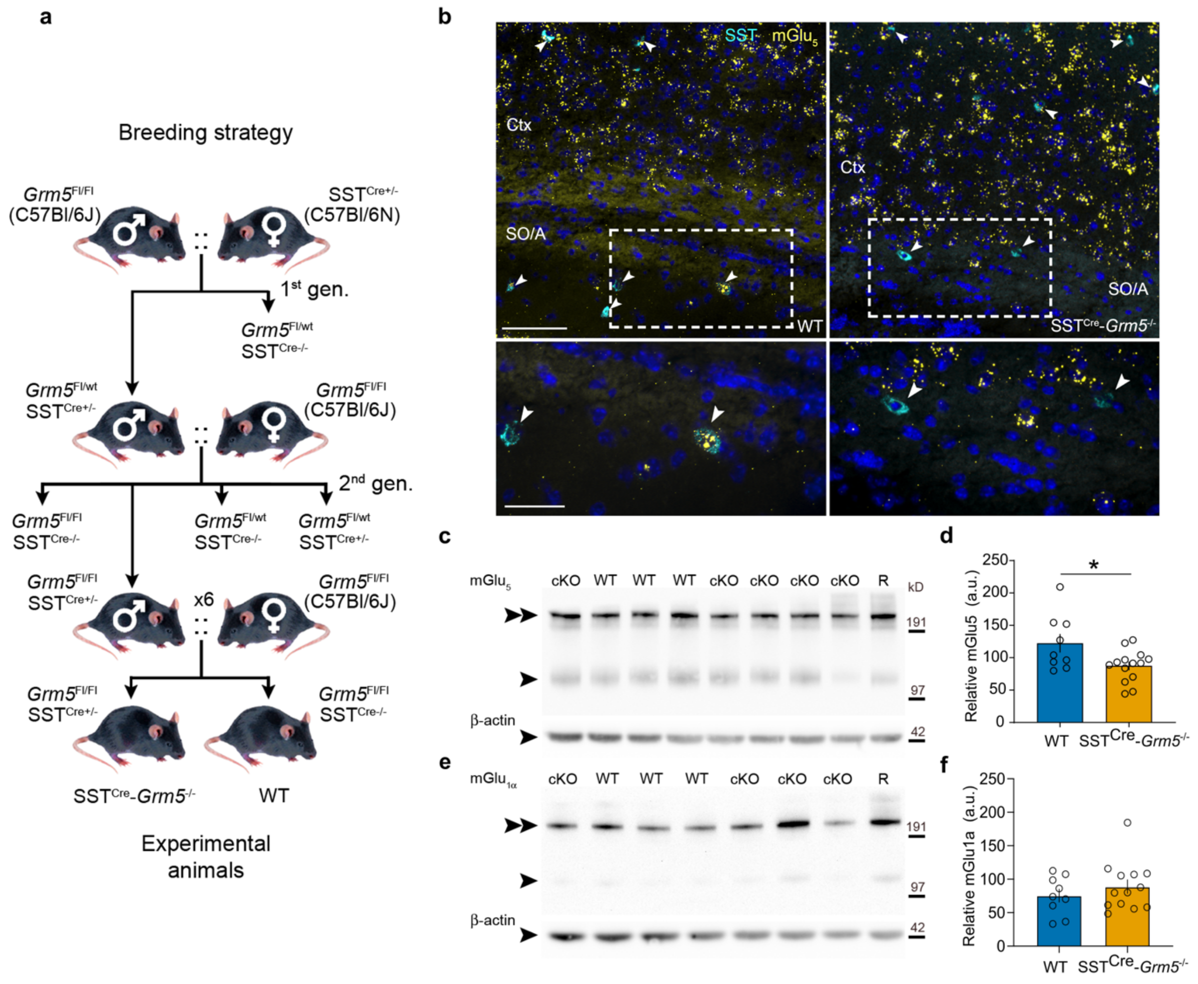

Suppl. Figure 3. Breeding strategy and characterization of SST^{Cre}-*Grm5*$^{-/-}$ mice.

(a) Schematics of the breeding strategy used to generate SST^{Cre}-*Grm5*-/- mice and WT littermates. **(b)** Representative examples of RNA staining via RNAscope of $mGlu_5$ and SST, in hippocampus and cortex, in WT (left panels) and SST^{Cre}-*Grm5*$^{-/-}$ mice (right panels). Scale bars: upper panels 100 µm; lower panels 50 µm. **(c-d)** SST^{Cre}-*Grm5*$^{-/-}$ mice display reduced expression of $mGlu_5$ as measured by western blot in the hippocampus (unpaired t-test, t_{21} = 2.50, p<0.05). **(e-f)** SST^{Cre}-*Grm5*$^{-/-}$ mice display a similar expression of $mGlu_{1a}$ as measured by western blot in the hippocampus (unpaired t-test, t_{135} = 0.95, p>0.05). Data are represented as mean ± SEM and individual data points. *p<0.05.

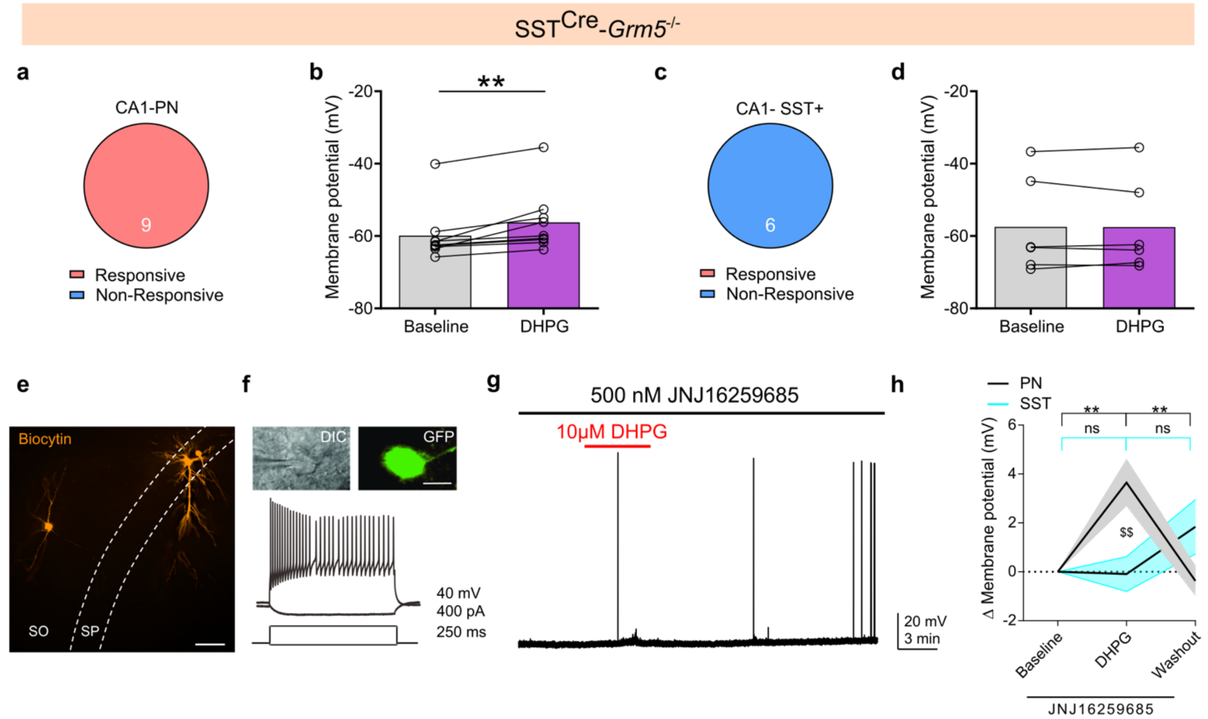

Suppl. Figure 4. Electrophysiological characterization of SST^{Cre}-*Grm5*$^{-/-}$ mice.

(a-b) All recorded CA1 PNs (9/9) in SST^{Cre}-*Grm5*$^{-/-}$ mice responded to DHPG infusion. **(b)** DHPG infusion significantly increased membrane potential (Wilcoxon matched-pairs rank test, W=45) in PNs. **(c-d)** In CA1, recorded SST+ INs (6/6) did not respond to DHPG infusion. Plots in (**b**,**d)** represent mean and individual values, **p<0.01. **(g)** Example of Biocytin-filled CA1 PNs (right) and SST+ IN (left) with its respective **(f)** DIC image and membrane responses to current injections. **(g)** Example trace displaying the lack of membrane depolarization upon DHPG infusion in the presence of the mGlu$_1$ antagonist JNJ16259685 in the SST+ IN shown in (**e**). **(h)** Quantification of DHPG-mediated membrane potential change in PNs and SST+ INs in the presence of JNJ16259685 (two-way ANOVA, Genotype $F_{1,13} = 0.70$, $p>0.05$; Treatment $F_{2,26} = 3.34$, $p>0.05$; Interaction $F_{2,26} = 9.45$, $p<0.001$; followed by Bonferroni multiple comparisons test: within neuron type **$p<0.01$; within treatment type $$p<0.01). Data are shown as mean ± SEM.

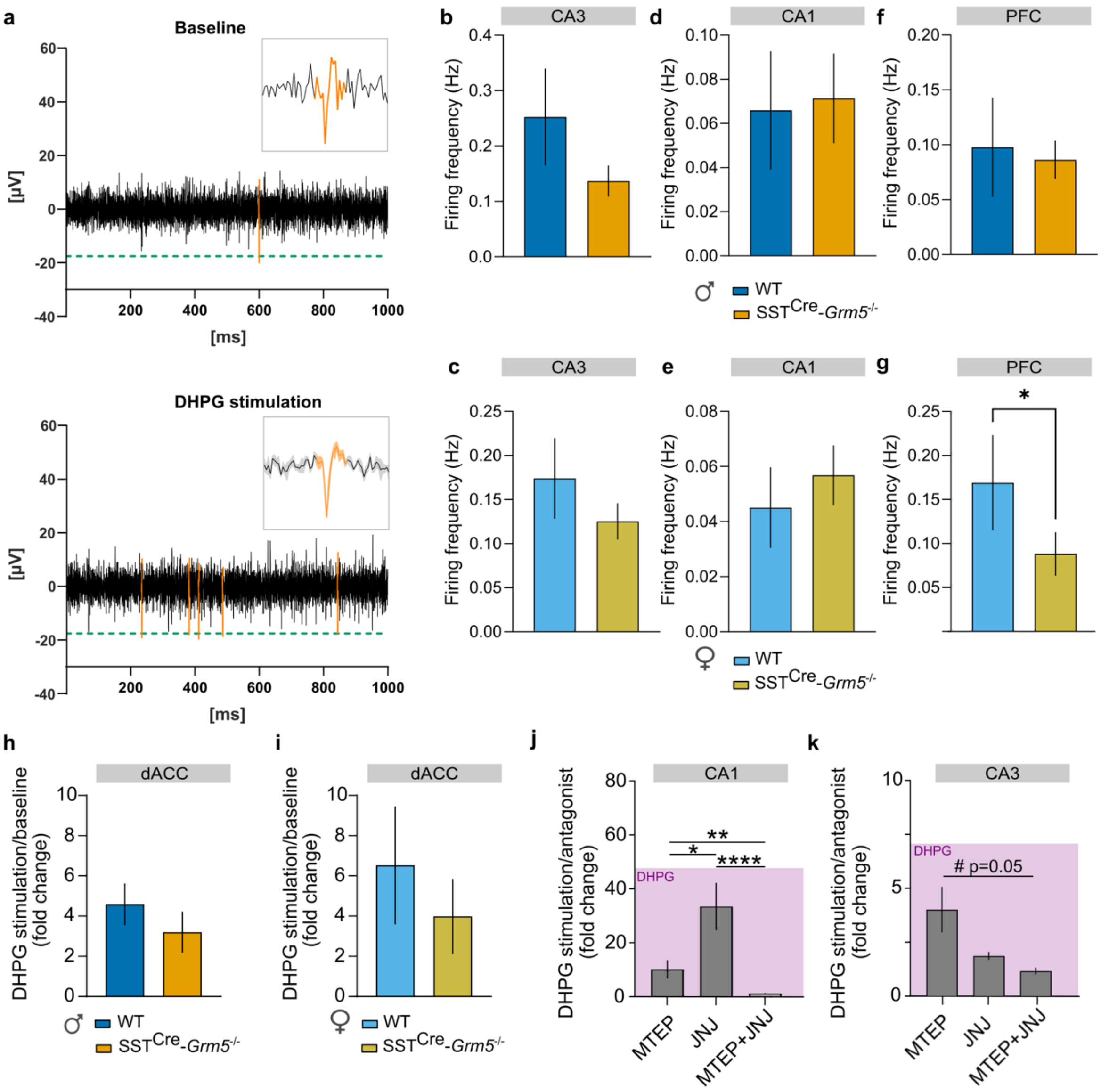

Suppl. Figure 5. Contribution of mGlu5 in SST+ neurons to network activity.

(a) Example traces including spike waveforms for baseline and following DHPG stimulation recorded in the CA3 subfield in WT mice. **(b-g)** Average baseline frequencies measured during MEA recordings in the **(b-c)** CA3, **(d-e)** CA1 and **(f-g)** PFC of WT and SST^{Cre}-*Grm5*$^{-/-}$ male and female mice. A decreased baseline firing frequency (Hz) was observed in the PFC of female mice only (Mann-Whitney U = 337, *p<0.05). **(h-i)** Fold change upon DHPG stimulation in the dACC was unchanged in SST^{Cre}-*Grm5*$^{-/-}$ compared to WT male (**h**; unpaired t-test, t_{135} = 0.95, p>0.05) and female (**i**; Mann-Whitney U = 913, p>0.05) mice. **(j-k)** DHPG stimulation/antagonist fold change was inhibited to a greater extent by MTEP compared to

JNJ16259685 in the male **(j)** CA1 region of the hippocampus; MTEP and JNJ16259685 showed an additive effect and completely abolished the DHPG-mediated activity (Kruskal-Wallis test, K = 29.85, ****p<0.0001; followed by Dunn's multiple comparisons test *p<0.05, **p<0.01, ****p<0.0001). These differences were absent in the CA3 region **(k)** (Kruskal-Wallis test, K = 6.10, *p<0.05; followed by Dunn's multiple comparisons test: MTEP vs JNJ p>0.99; JNJ vs MTEP+JNJ p=0.12; MTEP vs MTEP+JNJ p=0.05). The colored background represents fold change upon DHPG application only. Data are represented as mean ± SEM.

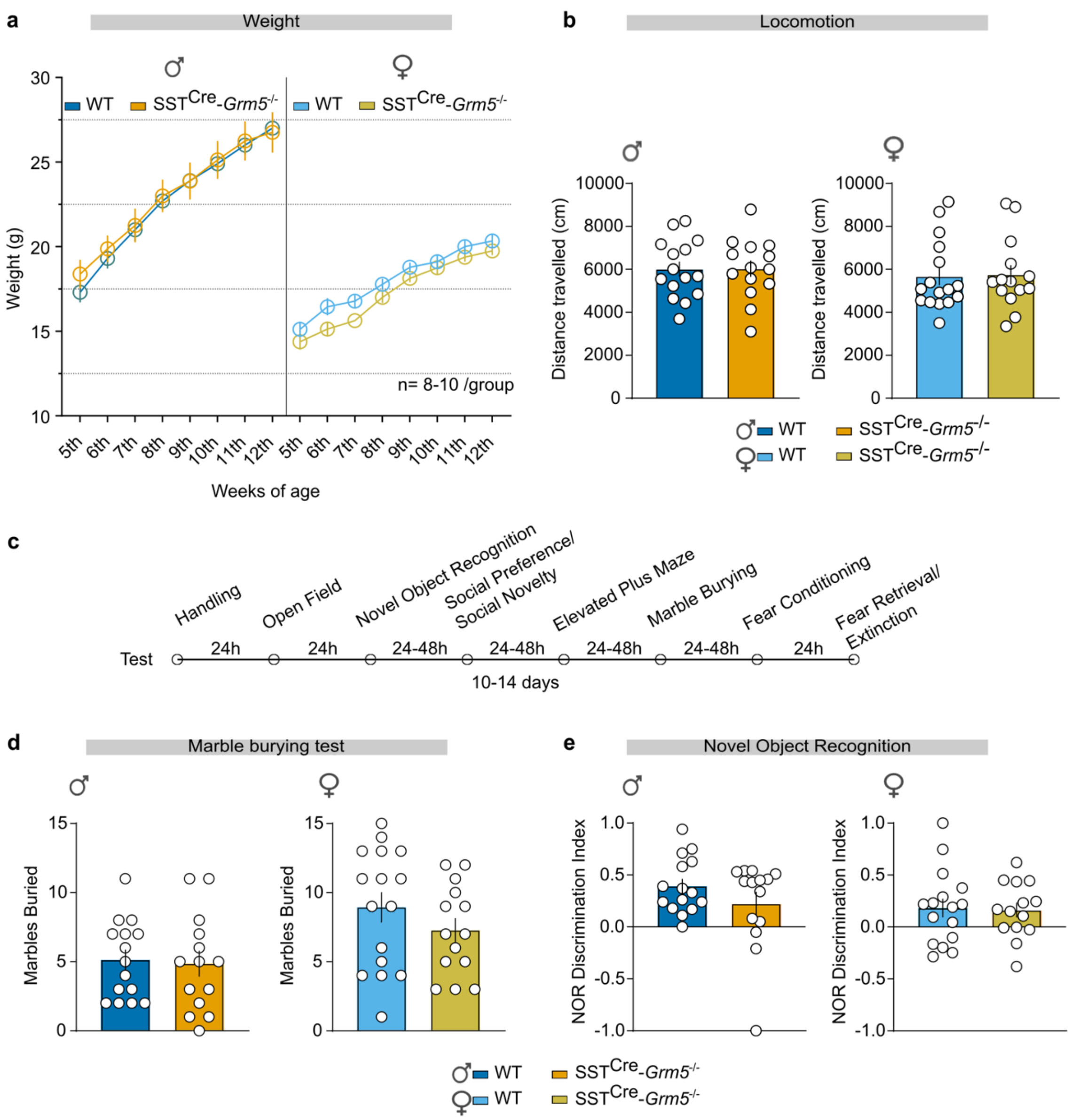

Suppl. Figure 6. SST^{Cre}-*Grm5*$^{-/-}$ mice have normal weight, locomotion, compulsive-like behavior and recognition memory.

(a) Weight curves in WT and SST^{Cre}-*Grm5*$^{-/-}$ animals were comparable from post-weaning until adulthood (Males: two-way ANOVA, Genotype $F_{1,16} = 0.08$, $p>0.05$; Females: two-way ANOVA, Genotype $F_{1,15} = 3.31$, $p>0.05$). **(b)** During a 20 min session of a non-anxiogenic low-lux (20Lux) Open Field exploration, WT and SST^{Cre}-*Grm5*$^{-/-}$ animals travelled similar distances (Males: unpaired t-test, $t_{27} = 0.04$, $p>0.05$; Females: unpaired t-test, $t_{28} = 0.13$, $p>0.05$). **(c)** Behavioral tests included in the behavioral phenotyping of SST^{Cre}-*Grm5*$^{-/-}$ mice. Tests were performed sequentially, and spaced with either 24 or 24-48 hours. **(d)** WT and SST^{Cre}-*Grm5*$^{-/-}$

mice showed similar levels of obsessive-compulsive-like behavior as measured by marbles buried in a marble burying test (Males: Mann-Whitney, $U = 95.5$, $p>0.05$; Females: Mann-Whitney, $U = 83$, $p>0.05$). **(e)** Similarly, no differences were observed in novel object recognition between WT and SST^{Cre}-*Grm5*$^{-/-}$ animals (Males: unpaired t-test, $t_{27} = 1.29$, $p>0.05$; Females: unpaired t-test, $t_{28} = 0.17$, $p>0.05$). Data are shown as mean ± SEM and individual data points.

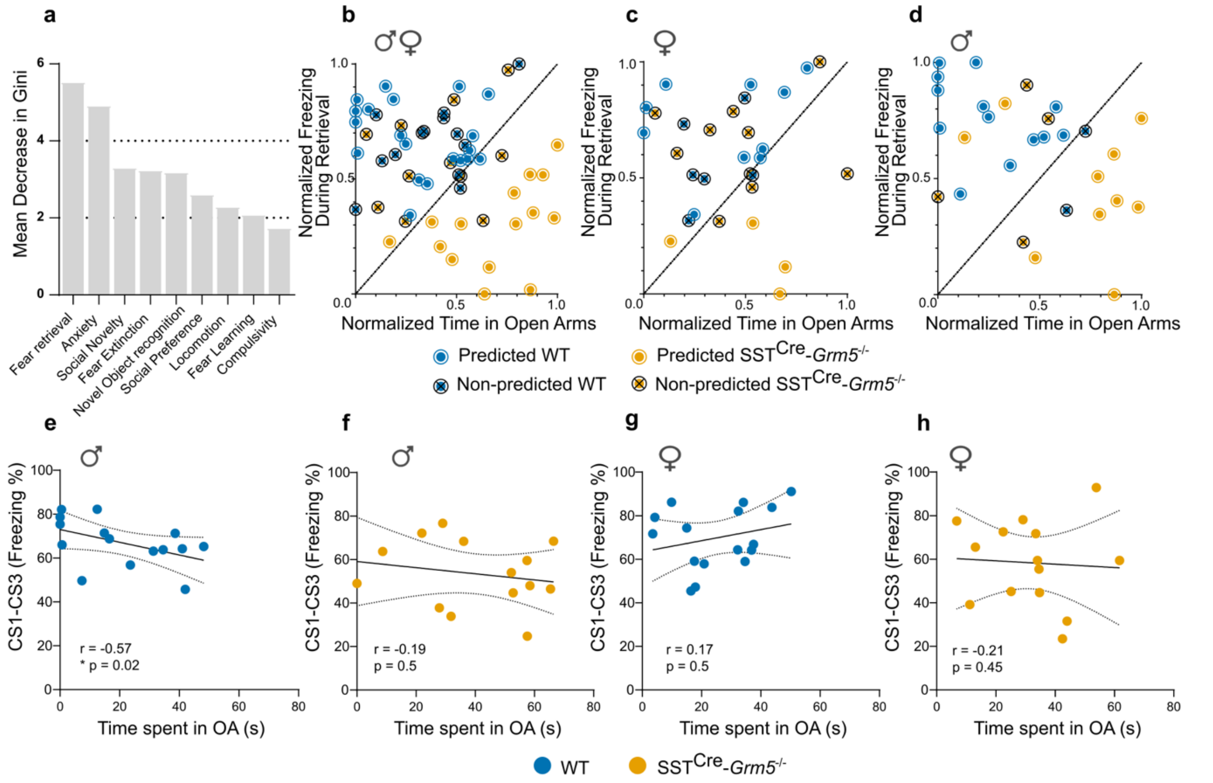

Suppl. Figure 7. Discriminating classification of WT and SST^{Cre}-*Grm5*$^{-/-}$ mice

(a) Gini index for each behavioral trait used for the random forest classification. **(b-d)** RF classification for the two most discriminating variables in all mice regardless of sex (**b**), females (**c**) and males (**d**).

(e-h) Spearman correlations between freezing during fear retrieval (Cs1-Cs3) and anxiety-like measures (Time spent in Open Arms). Only in WT male mice, fear retrieval and anxiety were significantly correlated. *p<0.05.

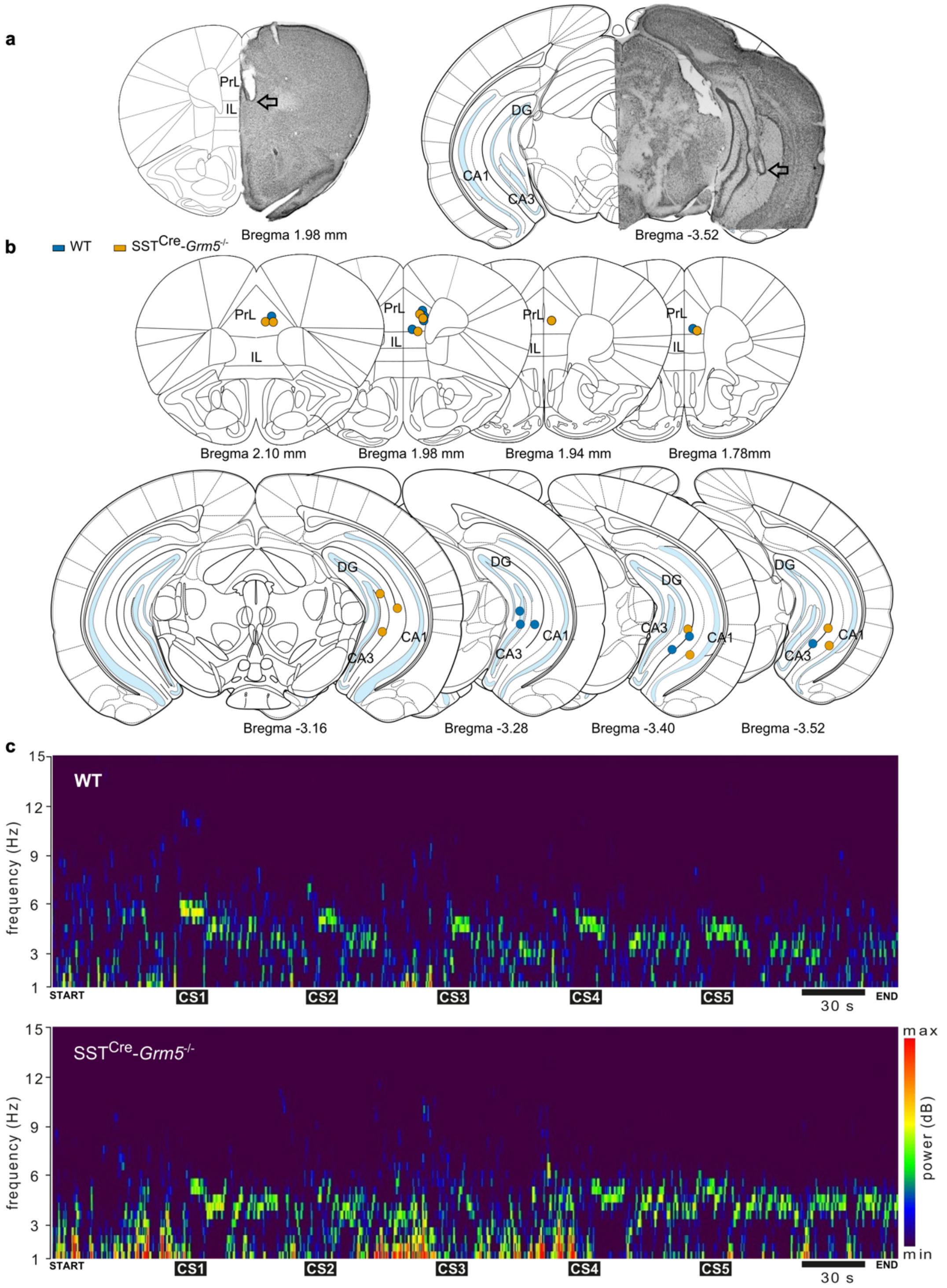

Suppl. Figure 8. Implant verifications and LFP recording during Fear Retrieval.

(a) Representative histological verification of unilateral LFP recording probe implanted in the mPFC and vHPC and **(b)** implantation site per animal and brain region. **(c)** Sonograms of entire LFP recordings of a representative WT and SST^{Cre}-*Grm5*$^{-/-}$ mouse during fear retrieval.

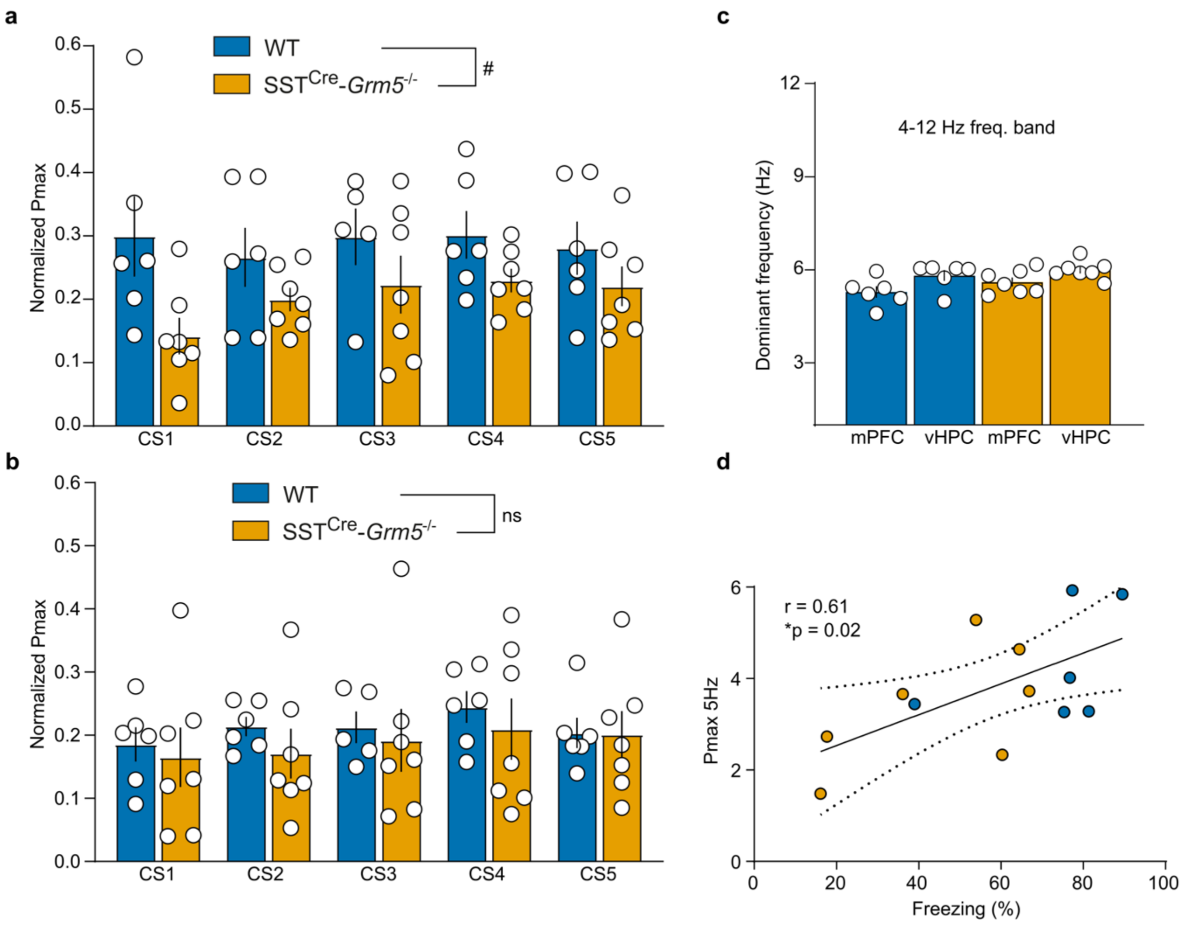

Suppl. Figure 9. Signal peak power and dominant frequency during Fear Retrieval in WT and SST^Cre^-*Grm5*^-/-^ mice.

(a-b) Signal peak power in theta frequency band (Pmax) during CS1-CS5 presentations in the **(a)** mPFC (two-way ANOVA, Genotype $F_{1,11} = 3.89$, $p>0.05$; Interaction, $F_{4,44} = 1.36$, $p>0.05$) and (**b**) vHPC (two-way ANOVA, Genotype $F_{1,11} = 0.34$, $p>0.05$; Interaction, $F_{4,44} = 0.24$, $p>0.05$). **(c)** The dominant frequency in the theta frequency band was comparable throughout areas and genotypes (two-way ANOVA, Genotype $F_{1,11} = 2.72$, $p>0.05$; Interaction, $F_{1,11} = 0.23$, $p>0.05$). **(d)** Correlation between the 5 Hz (peak frequency of the LFP signal in the theta band) signal power in mPFC and amount of freezing expressed during all 5 CS presentations in the fear retrieval session. $^{\#}$p =0.07, **p<0.01.

SUPPLEMENTARY TABLES

Suppl. Table 1. Summary of the sources and dilutions of primary and secondary antibodies

Primary	Source	Species	Dilution	Secondary/Dilution	Procedure
mGlu5	Frontier Institute Co.; cat. No. mGluR5-Rb-Af300	Rabbit polyclonal	1:500	Jackson Immuno Res.; Dnk anti-Rb Cy3 (1:500); cat. No. 711-165-152<br>-<br>Invitrogen; Dnk anti-Rb Alexa Fluor-488™; (1:1000); cat. No. A21206	IF
mGlu5	Frontier Institute Co.; cat. No. mGluR5-GP-Af270	G. Pig polyclonal	1:500	Jackson Immuno Res.; Dnk anti-G. pig Cy3 (1:500); cat. No. 706-165-148<br>-<br>Nanoprobes; Nanogold-goat anti G. pig Fab' (1:100) cat. No. 2055	IF<br>EM
mGlu5	Frontier Institute Co.; cat. No. mGluR5-Go-Af540	Goat polyclonal	1:500	Jackson Immuno Res.; Dnk anti-goat Cy3 (1:500); cat. No. 705-165-147	IF
PKCδ	BD Bioscience; cat. No.610397	Mouse monoclonal	1:500	Jackson Immuno Res.; Dnk anti-mouse Cy3; (1:500) cat. No. 715-165-150	IF
PV	Synaptic Systems; cat. No. 195 004; Lot. No. 195004/1	G. Pig polyclonal	1:500	Jackson Immuno Res.; anti-G. pig Dylight 405; (1:500); cat. No. 706-475-148	IF
SST	Kindly provided by Prof. G. Sperk (Dept. Pharmacol., Innsbruck, Austria)	Rabbit polyclonal	1:1.000	Invitrogen; Dnk anti-Rb Alexa Fluor-488™;(1:1000); cat. No. A21206<br><br>Vector; biotinylated goat anti Rb; (1:100); cat. No. BA-1000	IF<br>EM
SST	Santa Cruz; cat. No. sc-7819	Goat polyclonal	1:500	Invitrogen; Dnk-anti goat Alexa Fluor-488™; (1:1000); cat. No. A11055	IF

Suppl. Table 2. Colocalization between $mGlu_5$ and SST or PKCδ in neurons

Brain area	Co-expression (%)	Double-labeled	SST+ Cells analyzed (n)	PKCδ+ Cells analyzed (n)
**Neocortex**				
Sensory (SS-V1-2)	68.2	15	22	
PFC	80.5	12	15	
**Hippocampus**				
CA1 subfield	84.4	38	45	
**Amygdala**				
BLA	55.0	11	20	
CeA	0.01	1	116	
	91,0	31		34
**Other areas**				
LS	0.0	0	36	

Colocalization was assessed from confocal z-stacks taken from at least 2 sections/animal (n=3).

SUPPLEMENTARY MATERIALS AND METHODS

Western Blotting

Membranes were prepared according to a previously published protocol (Romano et al., 1995). In brief, both hippocampi from each animal were pooled and homogenized in ice-cold 10 mM Tris-HCl, pH 7.4 buffer containing 320 mM sucrose, 1 mM phenylmethylsulphonyl fluoride, 1 mM NaF, 1 mM Na_3VO_4 and complete EDTA-free protease inhibitors (Roche, Vienna, Austria) using a motorized homogenizer (Sartorius) at a speed of 1,400 rpm and 10 strokes. Lysates were centrifuged for 10 min at 1,000 x g, 4 °C to remove unbroken cells and tissues. This process was repeated 2 more times. Supernatants were collected and centrifuged for 40 min at 17,000 x g, 4 °C. The pellet (P2 fraction) was resuspended in ice-cold 25 mM Tris-HCl, pH 7.4 buffer containing 1 mM NaF, 1 mM Na_3VO_4 and complete EDTA-free protease inhibitors. Protein concentration was determined by Bradford protein assay using bovine serum albumin as the standard protein.

P2 samples were denatured in Laemmli sample buffer containing 30 mM dithiothreitol and heated for 5 min at 60°C, to prevent oligomerization, for sodium dodecylsulphate-polyacrylamide gel electrophoresis (SDS-PAGE). Samples were loaded (20 μg) on NuPAGE Bis-Tris 4-12% precast gels and proteins were resolved at 80 V in MOPS SDS buffer. Proteins were transferred to polyvinylidene difluoride membrane overnight at 150 mA, 6 °C. The membranes were stained with Ponceau-S for 10 min and cut at approximately 80 kDa. They were then incubated in 5% dry milk blocking solution for 1 h at room temperature. The half containing the high molecular weight proteins was probed with a rabbit polyclonal antibody raised against $mGlu_5$ (Frontier Sciences, Sapporo, Japan; cat. No. mGluR5-Rb_Af300; diluted 1:3000) or with a rabbit polyclonal antibody raised against $mGlu_1$ (Frontier Sciences, Sapporo, Japan; cat. No. mGluR1-Rb_Af811; diluted 1:3000), whereas the half containing the low molecular weight proteins was incubated with a rabbit polyclonal antibody raised against β-

actin (Sigma-Aldrich cat. No. A2228; diluted 1:3000) for 3 overnights at 6°C with constant rocking. Immunoreactive bands were detected by incubation in horseradish peroxidase-conjugated secondary antibodies (Invitrogen) followed by the ECL Prime reagent. Chemiluminescence was visualized with the Fusion SL-4 Vilber Lourmat imaging system (Peqlab, Erlangen, Germany). To control for interblot variations, each gel contained a reference internal tissue standard (P2 membranes prepared from the neocortex of control C57Bl/6j mice). Membranes from individual animals were loaded at least in three different and independent gels. Densitometric analysis was performed using the ImageJ gel analysis application.

Immunofluorescence experiments

Immunofluorescence experiments were performed according to previously described procedures (Sreepathi & Ferraguti, 2012; Zangrandi et al., 2021). Briefly, after blocking with Tris-buffered saline (TBS) complemented with 0.1% Triton X-100 and 20% normal serum (NS) for 1 h, the sections were incubated in primary antibodies (see Suppl. Table 1 for complete listing and dilutions) in a solution of 2% NS, 0.1% Triton X-100 in TBS (pH 7.4) for 48 h at 6° C. All primary antibodies against $mGlu_5$ have been tested on sections from germline *Grm5* KO animals and produced no immunoreactive signal (Uchigashima et al., 2007)(personal communication). After multiple washes in TBS, the sections were then incubated overnight with the respective secondary antibodies (see Suppl. Table 1). After three washing steps with TBS, the sections were finally mounted onto gelatin-coated slides and coverslipped with Vectashield (Vector Laboratories, Burlingame, CA, USA). Immunofluorescence was first examined in epifluorescence using a Zeiss AxioImager M1 microscope (Carl Zeiss, Oberkochen, Germany). Confocal microscopy was performed using an Airy Scan LSM980 laser scanning microscope (Carl Zeiss) with a 63x/1.2 NA objective or a Leica TCS SP8 gSTED microscope equipped with an HCX PL APO 63x/1.3 NA objective (Leica Microsystems). Raw images were channel dye separated and deconvolved using the Huygens software (Scientific

Volume Imaging, Hilversum, The Netherlands). Z-stacks were analyzed using the IMARIS 9.7.0 software (Oxford Instruments, Bitplane, Zurich, Switzerland). To identify a neuron as immunoreactive for $mGlu_5$, at least 10 consecutive sections had to contain labeled adjoining parts of the plasma membrane.

In Situ Hybridization RNAscope

Coronal sections 12 μm thick were cut on a cryostat, collected on slides and processed using the RNAscope fluorescent multiplex reagent kit (ACD) as described in (Fagan et al., 2020) and following the manufacturer's recommendations. The following probes were used for SST and $mGlu_5$: Mm-Grm5-C2 (target region 2409-3336; cat. no. 423631C2) and Mm-Sst-C1 (target region 18-407; cat. no. 404631C1; Bio-techne Ireland Ltd, Dublin, Ireland). Images of hippocampal sections subjected to RNAscope staining were acquired using an epifluorescence Zeiss AxioImager M1 microscope (Carl Zeiss, Oberkochen, Germany).

Pre-embedding immuno-electron microscopy

Immunocytochemistry for electron microscopy was performed as previously described (Dobi et al., 2013) and using similar conditions as used for the immunofluorescence experiments with the exception of omitting Triton X-100 from the buffers. To facilitate antibody penetration, the sections were first cryoprotected in 20% sucrose made in 0.1 M PB overnight at 6° C and then freeze-thawed twice after removal of the sucrose. After incubation in the primary antibodies (see Suppl. Table 1), the sections were extensively washed with TBS and then incubated overnight (6°C) in secondary antibodies (see Suppl. Table 1). In double labeling studies, $mGlu_5$ was visualized by silver-intensified immunogold reaction and SST by horse radish peroxidase reaction. After three washes in TBS, the sections were washed in double-distilled water, followed by silver enhancement of the gold particles with an HQ Silver kit (Nanoprobes) for 8–15 min. Sections were subsequently incubated in ABC complex (diluted 1:100; Vector

Laboratories) in TBS overnight at 6°C. Peroxidase was visualized with 3-3′-diaminobenzidine (0.5mg/mL) using 0.01% H_2O_2 as substrate for 5–10 min. After several washes in 0.1M PB, sections were then treated with 2% OsO_4, contrasted with 1% uranyl-acetate, dehydrated and embedded in epoxy resin (Durcupan ACM, Fluca; Sigma-Aldrich, Gillingham, UK). Serial ultrathin (70- to 80-nm-thick) sections were collected on pioloform-coated copper grids and analyzed in a transmission electron microscope (Philips CM120, Eindhoven, Netherlands). Digital micrographs were taken using a 11 Megapixel side-mounted TEM CCD-camera (Morada; SIS, Olympus, Münster, Germany).

Surgical procedures for patch-clamp recordings

Anesthesia was induced with a combination of intraperitoneally injected Ketamine (80 mg/kg; Ketasol, AniMedica) and Xylazine (5 mg/kg; Xylasol, Animedica) and maintained with 2% Sevofluran (SEVOrane). The head was then fixed on a stereotactic frame (Model 1900; Kopf Instruments) and ophthalmic ointment was applied to the eyes to avoid drying. SST^{Cre}-$Grm5^{-/-}$ male mice (n = 5) were unilaterally injected with the viral vector AAV2/5.CAG.flex.GFP (University of North Carolina vector core, UNC) into the hippocampus (coordinates from bregma: AP: -3.50 mm; ML: 3.50 mm; DV: 3.7 to 3 mm) in a volume of 1 µL. To allow for sufficient GFP expression, mice were sacrificed for patch-clamp recordings 3 weeks following the surgical procedure. Postoperative pain medication after surgery included administration of meloxicam (Metacam, Boehringer Ingelheim; 1 mg/kg subcutaneously).

Extended description of slice preparation and patch-clamp recordings

Animals at 2-3 months of age were sacrificed by rapid decapitation with isoflurane anesthesia. The dissected brain was mounted on the slicing chamber with oxygenated (95% O_2 and 5% CO_2) ice-cold sucrose saline containing (in mM): 87 NaCl, 25 $NaHCO_3$, 1.25 NaH_2PO_4, 2.5 KCl, 10 glucose, 75 sucrose, 0.5 $CaCl_2$, and 7 $MgCl_2$. Acute horizontal brain slices of 350

μm thickness were cut using a vibratome (DTK-1000; Dosaka, Kyoto, Japan). Slices were recovered in the oxygenated sucrose saline containing chamber at 34°C for 30 min and were then kept at room temperature until use. Neuronal recordings were performed in slices from n = 8 mice (7 females and 1 male) for all data presented in Fig. 1 and in slices from n = 5 males for all data presented in Suppl. Fig. 4.

During the electrophysiological experiments, slices were transferred to a submerged chamber and perfused with the oxygenated artificial cerebrospinal fluid (aCSF) at room temperature (23 ± 2°C). The aCSF used in *ex vivo* patch-clamp experiments was composed of (in mM): 125 NaCl, 25 $NaHCO_3$, 1.25 NaH_2PO_4, 2.5 KCl, 25 glucose, 2 $CaCl_2$, and 1 $MgCl_2$. The tdTomato or GFP expression in neurons was confirmed by epifluorescence. SST+ neurons in the CA1 and neocortex were visually selected for recordings under an infrared differential interference contrast microscope (IR-DIC; BX51WI, Olympus, Tokyo, Japan). During the *ex vivo* whole-cell patch-clamp recording, bath application of CNQX (10 μM; Tocris Bioscience, Bristol, UK) was used to block AMPA receptors, while gabazine (1 μM; Abcam, Cambridge, UK) and CGP55845 (1 μM; Tocris Bioscience) were used to block $GABA_A$ receptors and $GABA_B$ receptors, respectively. To examine the effect of $mGlu_5$ activation on SST+ neurons, the group I mGlu agonist (S)-3,5-dihydroxyphenylglycine (DHPG; 10 μM, Tocris Bioscience) and NAM 3-((2-Methyl-1,3-thiazol-4-yl)ethynyl)pyridine (MTEP; 10 μM, Tocris Bioscience) were applied alone or together with synaptic blockers via bath superfusion. In some experiments, 3,4-Dihydro-2H-pyrano[(2,3-b])quinolin-7-yl)-(cis-4-methoxycyclohexyl)-methanone (JNJ16259685 (500 nM; Tocris Bioscience) was bath-applied to block $mGlu_1$.

Whole-cell patch-clamp recordings were made using the Axopatch 200B amplifier or Multiclamp 700B amplifier (Molecular Devices, San Jose, CA, USA). Recording electrodes (3–7 MΩ) were pulled from borosilicate glasses (outer diameter, 1.5 mm; 0.32 mm wall thickness; Harvard Apparatus, Holliston, MA, USA) and filled with a low Cl^- internal solution, which contained (in mM): 136.8 K-gluconate, 7.2 KCl, 0.2 EGTA, 4 MgATP, 10 HEPES, 7

Na_2-phosphocreatine, 0.5 Na_3GTP (pH 7.3 with KOH), and 0.4% biocytin (wt/vol, Thermo Fisher Scientific, Waltham, MA, USA). The pipette capacitance was compensated in the cell-attached mode. The series resistance was compensated to 100% in the current-clamp configuration. Signals were low-pass filtered at 4 kHz (four-pole Bessel filter) and sampled at 10k Hz using a digitizer (Digidata 1440A). Pulse sequences were generated by pClamp 10.7 (Molecular Devices). Electrophysiological data were analyzed using Clampfit 10.7 (Molecular Devices). During the membrane potential (MP) recording in the current clamp mode, a constant negative current was injected to maintain the spontaneous firing rate below 1 Hz during baseline. The passive recording was performed if the spontaneous firing rate of the cell did not exceed 1 Hz. To measure the MP change before and after drug applications, the recorded traces were low-pass filtered with a cutoff frequency of 3 Hz. The averaged MP of the last 1 min of each treatment was detected.

Histology and imaging of biocytin-filled neurons

To identify the biocytin-filled neurons, brain slices were fixed overnight with 4% PFA (wt/vol) in phosphate-buffered saline (PBS). After washing three times with PBS, slices were soaked in 0.3% Triton X-100 (vol/vol; USB Co., Cleveland, OH, USA) for 30 min. The slices were then incubated with Alexa Fluor® 488 streptavidin (1:400; Thermo Fisher Scientific, Waltham, MA, USA) in PBS containing 0.3% Triton X-100 and 2% normal goat serum (NGS, Vector Laboratories) at 4°C overnight or at room temperature for 2 h. After washing six times with PBS, slices were mounted onto slides using Vectashield mounting medium containing 4′,6-diamidino-2-phenylindole (DAPI, H-1200, Vector Laboratories). Biocytin-labeled cells, tdTomato, GFP and DAPI signals were imaged using a confocal laser excitation microscope Leica SP5 (Leica Microsystems).

Behavioral phenotyping

SST^{Cre}-$Grm5^{-/-}$ mice showed no overt phenotypic alterations in development assessed with the Irwin's test, nor macroscopic anatomical abnormalities. Both male and female mice were used in this project. All experiments were performed during the light cycle. Only mice aged 10-18 week old were used for behavioral experiments. Prior to all experiments, animals were handled for a minimum of three days and acclimatized to the testing rooms for behavioral phenotyping for at least 24 h. Following the last behavioral test, female mice underwent a vaginal swap, and all mice were either culled or perfused for immunohistochemical studies. Estrous stage assessment was performed as previously described (Pantier et al., 2019). All animals used in this study, besides an extra cohort of females tested only in the fear conditioning and extinction paradigm, performed all tests with no exception (14-16 mice per genotype of each sex). In order to have the minimum carry-on effect of one test onto the other, anxiogenic and stressful tests (e.g. elevated plus maze marble burying, fear conditioning and retrieval) were performed the latter, respectively, as described in Suppl. Fig. 6.

Social preference and social novelty

The social preference indexes were calculated as following:

$$\frac{Time\ spent\ in\ social\ interaction\ zone - Time\ spent\ in\ object\ \ interaction\ zone}{Total\ time\ spent\ in\ interaction\ zones} x100$$

The social novelty indexes were calculated as following:

$$\frac{Time\ spent\ in\ novel\ mouse\ interaction\ zone - Time\ spent\ in\ object\ \ interaction\ zone}{Total\ time\ spent\ in\ interaction\ zones}\ \mathrm{x100}$$

Open field and novel object recognition

On day 1, each mouse was placed in a square open field arena (50x50x35 cm) made of grey opaque plastic and allowed to explore it for 20 min. Distance travelled (in cm) was taken as an assessment of locomotion. In order to avoid confounding effects of anxiety on locomotion, illumination was set at 30 Lux. On day 2, mice were placed in the same arena containing two identical objects (gray stone cylinders) for a 10 min familiarization trial. Object recognition memory was tested 1 h later during a 5 min discrimination trial in the arena containing a familiar and a novel object (Lego block). The arena and objects were cleaned with 70% ethanol between trials.

Each trial was recorded with a video camera mounted on top of the arena and time spent investigating the objects (<5cm from the object) was automatically scored using Ethovision XT 12 software (Noldus; RRID:SCR_000441). The discrimination ratio was calculated as following: $\frac{Time\ spent\ investigating\ novel-familiar\ object}{total\ time\ investigating} x100$.

Marble burying

Mice were individually placed in a clean standard type II cage filled with fresh 5 cm-deep sawdust, on top of which were positioned 15 small glass marbles arranged in five evenly spaced rows of three marble each. Testing was conducted for 20 min at 30 Lux and marbles were considered buried if at least 2/3 of the marble was covered with bedding.

Random forest analysis of behavior

A statistical model for global discrimination between genotypes was performed using random forest (RF) classification with leave-one out of bag validation and 500x sample permutations using the R package Classification and Regression by random forest (Liaw & Wiener, 2002). The behavioral traits included in this analysis were the variables from the following different behavioral tests: locomotion (distance travelled in an Open Field), novel object recognition

(object discrimination ratio), compulsivity (marbles buried), social preference (social preference ratio), social novelty (social novelty ratio), anxiety (time spent in open arms), fear learning (AUC of freezing during fear conditioning), fear retrieval (mean freezing during fear retrieval) and fear extinction (AUC of freezing during fear extinction). Missing values are not tolerated by the model, and thus, we did not perform outlier analysis on any of the aforementioned behavioral tests.

Extended description of MEA electrophysiological recordings *in vitro*

Brain slices were prepared from 12-17 week old mice as previously described (Mitrić et al., 2019). The animals were anesthetized with isoflurane (IsoFlo®, Zoetis) and decapitated. Brains were rapidly removed and immersed in ice-cold oxygenated (95% O2, 5% CO2) artificial cerebrospinal fluid (aCSF) with sucrose containing (in mM): sucrose 75, D–glucose 10, NaCl 87, NaHCO3 25, NaH2PO4 1.25, KCl 2.5, CaCl2 0.5 and MgCl2 7 (osmolarity: ~320 mOsm/kg, pH adjusted to 7.4 with HCl). The brains were then trimmed with a scalpel blade in sucrose aCSF and glued onto the stage of a vibrating microtome (VT1200S, Leica Microsystems, Germany) for preparation of coronal slices (300 µm) containing the hippocampal area. The slices were incubated in oxygenated sucrose aCSF at 32°C for 30 min and subsequently transferred to oxygenated standard aCSF containing (in mM): NaCl 125, NaHCO3 25, D–glucose 25, KCl 2.5, NaH2PO4 1.25, CaCl2 2 and MgCl2 1 (osmolarity: ~319 mOsm/kg, pH adjusted to 7.4 with HCl). The slices were kept in a holding chamber at room temperature for at least 1 h before starting with the electrophysiological recordings.

MEA recordings were performed on a MEA2100 recording system (Multi Channel Systems, Reutlingen, Germany) as previously described (Kummer et al., 2015). The position of the brain slices on the electrode field was documented on an inverted Leica DMi1 microscope (Leica Microsystems, Wetzlar, Germany) using a Leica MC120 HD digital microscope camera (Leica Microsystems) for subsequent analysis. The slices were transferred to planar MEA chips

(120MEA200/30iR-Ti, Multi Channel Systems) and a platinum slice grid spanned with nylon fibres was carefully put on top of the slices. The brain slices were superfused with oxygenated standard aCSF at 32-34°C during the recordings. After a short habituation period, spontaneous action potential firing was recorded with a sampling rate of 5 kHz for 5 min. Some slices were pre-treated with the non-competitive $mGlu_5$ antagonist 3-((2-Methyl-1,3-thiazol-4-yl)ethynyl)pyridine (MTEP) (10 µM) and/or mGlu1 antagonist 3,4-Dihydro-2H-pyrano[(2,3-b])quinolin-7-yl)-(cis-4-methoxycyclohexyl)-methanone (JNJ 16259685) (1 µM) for 10 min. The glutamatergic stimulation of the hippocampus was induced by applying the non-selective group I metabotropic glutamate receptor agonist (S)-3,5-dihydroxyphenylglycine (DHPG) (50 µM) for 2 min. The recorded traces were analysed using the MC_Rack software (Multi Channel Systems). All data streams were filtered using a 200 Hz high-pass-filter. Spike detection was performed with a threshold of -5 standard deviations (SD) from noise. Electrodes were assigned to the respective brain regions according to the position of the electrodes on the images. Recording electrodes were classified as active if the spiking frequency exceeded 1/60 Hz (i.e., at least one AP per minute). Average spike frequencies were calculated for the different pharmacological treatments. Average spiking frequencies during baseline recordings and DHPG stimulation were calculated for each recording electrode separately, and the ratio "stimulation/baseline" calculated per electrode. Average ratios were used for statistical comparisons.

<u>LTP recordings</u>

For LTP (long-term potentiation) recordings, brains were removed and immersed in ice-cold oxygenated aCSF after decapitation. Brains were trimmed with a scalpel blade and a 15° piece was cut from the ventral part of the brain of each hemisphere (Bischofberger et al., 2006). One hemisphere was glued to the platform and the two hemispheres separated. Horizontal hippocampal slices (300 µm) were cut using a vibrating microtome (VT1200S, Leica

Microsystems) in ice-cold standard aCSF. The slices were transferred to a holding chamber filled with oxygenated standard aCSF at room temperature for at least 1h before start of the LTP recordings.

The slices were transferred to planar MEA chips (120MEA200/30iR-Ti, Multi Channel Systems) and positioned within the recording area to entirely cover the 12x12 array. Once the slices were fixed with a grid they were continuously perfused with oxygenated aCSF at a flowrate of 2-3 ml/min using a PPS2 peristaltic pump (Multi Channel Systems). For each LTP recording, the strength of the pulse was adapted to obtain field excitatory postsynaptic potentials (fEPSPs) with 50% maximal slope. Therefore, an initial input output (IO) curve with increasing amplitudes running from 500 mV to 4V was generated using the MC_Stimulus II software. The Schaffer collaterals were stimulated in the hippocampal region CA3 using 2-3 electrodes and the evoked fEPSPs located in CA1 area were detected. Biphasic constant pulses (0.1 ms /phase) were delivered every 60 s until a stable baseline was recorded for 15 min. LTP was induced by a theta burst stimulation (TBS) protocol (3 trains of 10 bursts at 5 Hz, 4 pulses at 100 Hz for each burst). Afterwards, test pulses were constantly delivered every 60 s for 40 min. The recordings were analyzed using the MC_Rack software (Multi Channel Systems). In order to quantify LTP, the fEPSP amplitudes after TBS stimulation were normalized to fEPSP amplitudes during baseline recording.

Extended description of electrophysiological recordings *in vivo*

Male mice (aged 2-3 months) were stereotactically (Kopf Instruments, USA) implanted under ketamine/xylazine combination (i.p) and sevoflurane (Sevorane, AbbVie GmbH, Austria) anesthesia, with recording electrodes made of twisted 76,2 um teflon coated, stainless steel wires (Science Products, Germany) into the mPFC (at 3° angle, AP:+1.8, L:+0.5, D:-1.7 mm – from bregma level) and ventral hippocampus (AP:-3.2, L:+3.3, D:- 2.8 mm from bregma level). A silver wire (Science Products, Germany) connected to a screw mounted posteriorly to bregma

was used as a ground/reference electrode. For additional support two small screws were also mounted to the skull. All electrodes were connected to a 10-pin PCB connector and cemented to the skull with dental acrylic (Paladur, Heraeus Kulzer GmbH, Germany). During the surgery ophthalmic ointment (to avoid eye drying) and postoperative pain medication - meloxicam (Metacam, Boehringer Ingelheim; 0.01 mg/kg subcutaneously) was applied. After 7-10 days of recovery time, animals were habituated to the LFP signal recording setup and fear conditioning chamber (4-5 sessions, 10 min/session, 1 session/day). LFP signals were recorded on EXT-9 recording system using headstage-commutator assembly (NPI electronic, Germany) allowing the animal for free movement inside the fear conditioning chamber. The row signal was amplified x1000, filtered 0.1–1 kHz, digitized 1kHz (Power 1401, CED, Cambridge, UK) and stored on a PC for offline analysis with the use of Spike-2 (ver. 8.08) software (CED, Cambridge, UK). Only animals positively verified regarding the signal quality (appropriate signal amplitude, no movement artifacts) were used for further experiments. Simultaneously with the EEG recording, the behavior of the animal was also video recorded for further analysis. LFP signals recorded from mPFC and vHPC during fear retrieval sessions were digitally (off-line) filtered (Butterworth second order high pass >1Hz and notch (50Hz) filters) and used for further analysis. For spectral analysis of the signal (1-75 Hz), Fast Fourier Transformation (FFT, resolution 512) was calculated for 9-15 artifacts-free 1s. Samples taken from each CS presentation, afterwards transformed (z- scored). Peak power (Pmax) in the theta frequency band (4-12 Hz) and corresponding dominant frequency was calculated (with Sudsa22.2S2 script). Pmax values obtained during each CS were afterwards normalized and compared between experimental groups. Additionally, the analysis of the signal power at the mean dominant frequency in the theta range (4-12 Hz) determined from the FFT was also correlated with the amount of freezing expressed by animals. Theta synchronization between mPFC-vHPC during tone (CS1-CS5) presentations was determined by calculating signal cross-correlation via the Spike-2 software of low-pass (16Hz) filtered waveforms. The level of the

second positive peak in the cross-correlogram (which correspond to the theta frequency peak) was quantified after alignment to the maximal positive peak, averaged across individual animals and statistically assessed.

References

Bischofberger, J., Engel, D., Li, L., Geiger, J. R., & Jonas, P. (2006). Patch-clamp recording from mossy fiber terminals in hippocampal slices. *Nat Protoc*, *1*(4), 2075-2081. https://doi.org/10.1038/nprot.2006.312

Dobi, A., Sartori, S. B., Busti, D., Van der Putten, H., Singewald, N., Shigemoto, R., & Ferraguti, F. (2013). Neural substrates for the distinct effects of presynaptic group III metabotropic glutamate receptors on extinction of contextual fear conditioning in mice. *Neuropharmacology*, *66*, 274-289. https://doi.org/10.1016/j.neuropharm.2012.05.025

Fagan, M. P., Ameroso, D., Meng, A., Rock, A., Maguire, J., & Rios, M. (2020). Essential and sex-specific effects of mGluR5 in ventromedial hypothalamus regulating estrogen signaling and glucose balance. *Proc Natl Acad Sci U S A*, *117*(32), 19566-19577. https://doi.org/10.1073/pnas.2011228117

Kummer, K. K., El Rawas, R., Kress, M., Saria, A., & Zernig, G. (2015). Social Interaction and Cocaine Conditioning in Mice Increase Spontaneous Spike Frequency in the Nucleus Accumbens or Septal Nuclei as Revealed by Multielectrode Array Recordings. *Pharmacology*, *95*(1-2), 42-49. https://doi.org/10.1159/000370314

Liaw, A., & Wiener, M. (2002). Classification and Regression by RandomForest. *R News*, *23*.

Mitrić, M., Seewald, A., Moschetti, G., Sacerdote, P., Ferraguti, F., Kummer, K. K., & Kress, M. (2019). Layer- and subregion-specific electrophysiological and morphological changes of the medial prefrontal cortex in a mouse model of neuropathic pain. *Scientific Reports*, *9*(1), 9479. https://doi.org/10.1038/s41598-019-45677-z

Pantier, L. K., Li, J., & Christian, C. A. (2019). Estrous Cycle Monitoring in Mice with Rapid Data Visualization and Analysis. *Bio Protoc*, *9*(17). https://doi.org/10.21769/BioProtoc.3354

Romano, C., Sesma, M. A., McDonald, C. T., O'Malley, K., Van den Pol, A. N., & Olney, J. W. (1995). Distribution of metabotropic glutamate receptor mGluR5 immunoreactivity in rat brain. *J Comp Neurol*, *355*(3), 455-469. https://doi.org/10.1002/cne.903550310

Sreepathi, H. K., & Ferraguti, F. (2012). Subpopulations of neurokinin 1 receptor-expressing neurons in the rat lateral amygdala display a differential pattern of innervation from distinct glutamatergic afferents. *Neuroscience*, *203*, 59-77. https://doi.org/https://doi.org/10.1016/j.neuroscience.2011.12.006

Uchigashima, M., Narushima, M., Fukaya, M., Katona, I., Kano, M., & Watanabe, M. (2007). Subcellular Arrangement of Molecules for 2-Arachidonoyl-Glycerol-Mediated Retrograde Signaling and Its Physiological Contribution to Synaptic Modulation in the Striatum. *The Journal of Neuroscience*, *27*(14), 3663-3676. https://doi.org/10.1523/jneurosci.0448-07.2007

Zangrandi, L., Schmuckermair, C., Ghareh, H., Castaldi, F., Heilbronn, R., Zernig, G., Ferraguti, F., & Ramos-Prats, A. (2021). Loss of mGluR5 in D1 Receptor-Expressing Neurons Improves Stress Coping. *Int J Mol Sci*, *22*(15). https://doi.org/10.3390/ijms22157826
